# Neutrophil Delivered Hollow Titania Covered Persistent Luminescent Nanosensitizer for Ultrosound Augmented Chemo/Immuno Glioblastoma Therapy

**DOI:** 10.1002/advs.202004381

**Published:** 2021-07-01

**Authors:** Yujie Li, Xucong Teng, Yongji Wang, Chunrong Yang, Xiuping Yan, Jinghong Li

**Affiliations:** ^1^ Department of Chemistry Key Laboratory of Bioorganic Phosphorus Chemistry & Chemical Biology Tsinghua University Beijing 100084 P. R. China; ^2^ State Key Laboratory of Food Science and Technology International Joint Laboratory on Food Safety Jiangnan University Wuxi 214122 China

**Keywords:** glioblastoma, multimodal therapy, persistent luminescent phosphor, titanium dioxide, ultrasound

## Abstract

Glioblastoma (GBM) is the most malignant brain tumor with unmet therapeutic demand. The blood‐brain‐barrier (BBB) and tumor heterogeneity limit the treatment effectiveness of various interventions. Here, an ultrasound augmented chemo/immuno therapy for GBM using a neutrophil‐delivered nanosensitizer, is developed. The sensitizer is composed of a ZnGa_2_O_4_:Cr^3+^ (ZGO) core for persistent luminescence imaging and a hollow sono‐sensitive TiO_2_ shell to generate reactive oxygen species (ROS) for controlled drug release. Immune checkpoint inhibitor (Anti‐PD‐1 antibody) is trapped in the interior of the porous ZGO@TiO_2_ with paclitaxel (PTX) loaded liposome encapsulation to form ZGO@TiO_2_@ALP. Delivered by neutrophils (NEs), ZGO@TiO_2_@ALP‐NEs can penetrate through BBB for GBM accumulation. After intravenous injection, ultrasound irradiation at GBM sites initiates ROS generation from ZGO@TiO_2_@ALP, leading to liposome destruction for PTX and anti‐PD‐1 antibody release to kill tumors and induce local inflammation, which in‐turn attractes more ZGO@TiO_2_@ALP‐NEs to migrate into tumor sites for augmented and sustained therapy. The treatment enhances the survival rate of the GBM bearing mice from 0% to 40% and endows them with long‐term immuno‐surveillance for tumor recurrence, providing a new approach for precision therapy against GBM and other cancers.

## Graphical abstract

Delivered by neutrophils (NEs), ZGO@TiO_2_@ALP‐NEs can penetrate through blood‐brain‐barrier for glioblastoma (GBM) accumulation. Ultrasound irradiation at GBM sites initiates generate reactive oxygen species generation from ZGO@TiO_2_@ALP, leading to liposome destruction for paclitaxel and anti‐PD‐1 antibody release to kill tumor and induce local inflammation, which in‐turn attracts more ZGO@TiO_2_@ALP‐NEs for augmented and sustained tumor elimination.

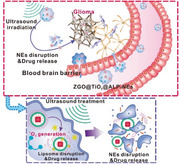

Glioblastoma (GBM) is the most malignant brain tumor with unsatisfied therapeutic outcome in clinic. Protected by the tight blood‐brain‐barrier (BBB),^[^ ^1^ ^]^ GBM is inert to various interventions (especially for the relative large‐scale multifunctional nanostructures^[^ ^2^ ^]^ and antibody treatments^[^ ^3^ ^]^) owing to the limited drug delivery and accumulation efficiency.^[^ ^4^ ^]^ In addition, traditional usage of non‐targeted small molecular drugs (such as temozolomide^[^ ^5^ ^]^) has evident side‐effects to the patients due to the high‐dosage systematic administration.^[^ ^6^ ^]^ Moreover, the complex microenvironment in GBM has high suppression for tumor killing and immune activation, making it difficult to avoid recurrence under single‐model therapy.^[^ ^7^ ^]^ Therefore, multi‐model therapeutic platforms with enhanced BBB penetration and GBM accumulation ability are preferable in GBM treatment.

Effective drug delivery is the foundation for GBM therapy.^[^ ^8^ ^]^ Various strategies such as surface modification,^[^ ^9^ ^]^ bionics^[^ ^10^ ^]^ and cell vehicles^[^ ^11^ ^]^ have been developed to improve the drug delivery efficiency across BBB.^[^ ^9^, ^12^ ^]^ As the most abundant leucocyte in blood, neutrophils (NEs) are promising carriers for intracranial therapy as they can respond to inflammation,^[^ ^13^ ^]^ adhere to and migrate across endothelial vessels (i.e., BBB^[^ ^14^ ^]^) into inflammatory tumor sites by shape change via intercellular route,^[^ ^15^ ^]^ making it possible to mediate BBB penetrable drug delivery for specifically GBM therapy.^[^ ^16^ ^]^ While, in order to maintain the above‐mentioned functions of the NEs vehicles, the NEs loaded therapeutic materials should be biocompatible during cell‐mediated transportations, that is to say, drugs should be accurately released in the target area (GBM) to prevent cytotoxic intra‐NEs leakage. Consequently, external stimulations are preferred to be introduced to achieve on‐demand treatment control.^[^ ^17^ ^]^ Between various activation sources, ultrasound shows great advantage in GBM therapy due to its deep penetration, non‐invasiveness and clinical availability.^[^ ^18^ ^]^ Titania (TiO_2_), the most common and biocompatible inorganic material, which could enhance the reactive oxygen species (ROS) generation under insonation,^[^ ^19^ ^]^ is suitable to act as sonosensitizers for controlled drug release in GBM treatment.^[^ ^20^ ^]^ Persistent luminescent materials, which have long‐time afterglow in the absence of real‐time excitation^[^ ^21^ ^]^ and can be re‐activated by red light‐emitting‐diode (LED),^[^ ^22^ ^]^ are ideal contrast agents for high signal to noise ratio (SNR) cell tracking,^[^ ^23^ ^]^ making it possible to visualize NEs transportation during therapeutic process.

Herein, to enhance GBM therapy with recurrence suppression, we designed a hollow TiO_2_ covered persistent luminescent nanosensitizer for optical imaging‐guided, ultrasound augmented chemo/immuno therapy against GBM. The nanosensitizer was composed of a ZnGa_2_O_4_:Cr^3+^ (ZGO) core^[^ ^22^ ^]^ for persistent luminescence imaging and a hollow TiO_2_ shell (as sonosensitizer) to ROS^[^ ^24^ ^]^ for therapy control. ZGO was a kind of persistent luminescent phosphor which shows near‐infrared (NIR)‐persistent luminescence with long afterglow time and renewability by red light‐emitting diode (LED) light.^[^ ^22^ ^]^ This unique optical property enables high signal‐to‐noise‐ratio (SNR) background‐free nanoplatform tracing, making it possible for precise GBM positioning to guide therapy. The porous ZGO@TiO_2_ was loaded with immue check point inhibitor anti‐PD‐1 antibody^[^ ^25^ ^]^ to relieve immunosuppression in GBM. Paclitaxel (PTX) loaded liposome was used as outermost layer of the matereial to realize chemo‐suppression against GBM acompanied with antibody encapsulation (**Figure** **1**a). The acquired ZGO@TiO_2_@ALP was internalized by NEs (ZGO@TiO_2_@ALP‐NEs) for BBB penetrable delivery (Figure 1b). After intravenous injection, the inflammation in GBM attracted ZGO@TiO_2_@ALP‐NEs to penetrate through the BBB via morphological change for GBM accumulation (Figure 1b). Ultrasound (US) irradiation at GBM sites initiated ROS generation from ZGO@TiO_2_@ALP to break up liposome coverage for PTX and anti‐PD‐1 antibody release to kill tumor and induce local inflammation, which in‐turn attracted more ZGO@TiO_2_@ALP‐NEs to tumor sites for augmented therapy against GBM.^[^ ^26^ ^]^

**Figure 1:**
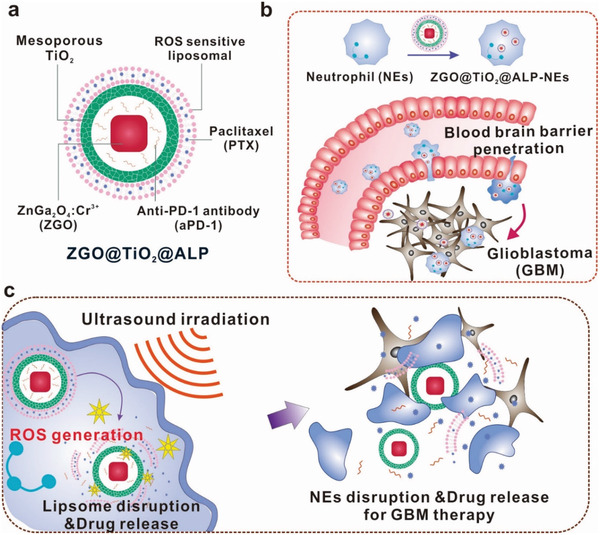
Schematic illustration of hollow TiO_2_ covered persistent luminescent nanosensitizer for ultrasound amplified chemo/immuno GBM therapy. a) Composition of ZGO@TiO_2_@APL. “A” represents anti‐PD‐1 antibody, “L” represents liposome, and “P” represents PTX in the abbreviation “ALP”. b) BBB penetration process of ZGO@TiO_2_@APL‐NEs. ZGO@TiO_2_@APL was loaded by neutrophils to form ZGO@TiO_2_@APL‐NEs in vitro. The injected ZGO@TiO_2_@APL‐NEs could be attracted by the inflammation in GBM to traverse the BBB. c) Ultrasound triggered drug release from ZGO@TiO_2_@APL‐NEs for GBM therapy. Upon insonation, ROS was generated from ZGO@TiO_2_@ALP to break up liposome coverage for PTX and anti‐PD‐1 antibody was released to kill tumor and induce local inflammation, which in‐turn attracted more ZGO@TiO_2_@ALP‐NEs to GBM sites for sustained therapy.

To realize high‐dosage antibody loading, controllable drug release, and clear optical imaging in one nanoplatform, we designed a hollow structure named ZGO@TiO_2_ as the fundamental component^[^ ^27^ ^]^ for further applications (Figure 1a; and Figures S1–S3, Supporting Information). The prepared ZGO@TiO_2_ had a 21 nm ZGO core with a 25 nm thick TiO_2_ shell (**Figure** **2**a; and Figure S2, Supporting Information), which showed strong ROS generation under insonation (Figure 2d; Figures S4 and S5, Supporting Information). The homogeneous mesoporous (pore size 4.5 nm) structures of ZGO@TiO_2_ (Figure S6, Supporting Information) provided possibility for anti‐PD‐1 antibody loading. ZGO@TiO_2_ had bright lasting near infrared (NIR) emission at 695 nm (Figure S7, Supporting Information). Prepared by annealing processes, ZGO@TiO_2_ were stable enough for notoxicitic in vivo applications (Figure S8, Supporting Information).

**Figure 2:**
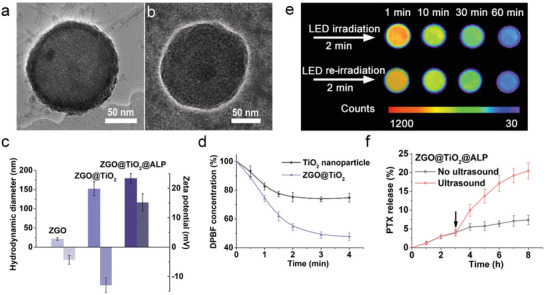
Characterization of ZGO@TiO_2_@APL. TEM image of a) ZGO@TiO_2_ and b) ZGO@TiO_2_@ALP. c) Size distribution and zeta potential of ZGO, ZGO@TiO_2_, and ZGO@TiO_2_@ALP. d) Time‐dependent ROS generation ability of ZGO@TiO_2_ under US irradiation (5 min, 1.5 MHz, 1.5 Wcm^−2^). e) Persistent luminescence image of ZGO@TiO_2_@ALP aqueous solution (100 µL, 0.3 mg mL^−1^). Persistent luminescence was activated following a 2 min red LED excitation (650 ± 10 nm) before imaging, and the signal acquisition time was set to be 120 s under the imaging system. f) In vitro PTX release from ZGO@TiO_2_@ALP with and without ultrasound irradiation. The arrows represent the application of ultrasound (5 min, 1.5 MHz, 1.5 W cm^−2^). Data are given as mean ± s.d. (*n* = 4).

Amino functionalization of ZGO@TiO_2_ induced manifest potential increase (from −12.9 to +3.1 mV) with negligible size variation (from 151.8 to 155.6 nm) (Figure S10, Supporting Information), enhancing the adsorbability of ZGO@TiO_2_ to antibody for immunotherapy. After anti‐PD‐1 antibody loading and PTX liposome encapsulation (Figure 2b), ZGO@TiO_2_@ALP showed 179 nm in size (Figure 2c) with a PTX encapsulation efficiency of 92.6%, PTX loading efficiency of 1.2% ,and anti‐PD‐1antibody loading content of 47 µg mg^−1^. ZGO@TiO_2_@ALP showed re‐activatable long‐lasting NIR afterglow after red LED light (650 ± 10 nm) irradiation (Figure 2e), making it possible for in vivo long‐term background free GBM tracing.^[^ ^22^ ^]^ ZGO@TiO_2_@ALP showed limited drug leakage under normal conditions (Figure S11, Supporting Information). While, ultrasound irradiation induced explosive PTX release from ZGO@TiO_2_@ALP (Figure 2f). Insonation triggered TiO_2_ shell to generate ROS, which decreased the stability of the liposomal bilayers by increasing the elastic modulus of the liposome, leading to the burst of drug release from ZGO@TiO_2_@ALP.^[^ ^24^ ^]^

ZGO@TiO_2_@ALP were internalized by NEs for targeted GBM transportation. ZGO@TiO_2_@ALP‐NEs were prepared by 2 h co‐incubation of the mouse‐isolated NEs (Figure S12, Supporting Information) with ZGO@TiO_2_@ALP. Compared with Taxol, ZGO@TiO_2_@ALP showed limited cytotoxicity towards NE even at high concentrations (Figure S13, Supporting Information), making it possible for NEs‐mediated material delivery.^[^ ^18^ ^]^ ZGO@TiO_2_@ALP‐NEs had a PTX loading capacity of 12 *μ*g per 10^6^ cells (Figure S14, Supporting Information). Limited PTX leaked from ZGO@TiO_2_@ALP‐NEs at physiological conditions while ultrasound treatment induced burst of PTX release from ZGO@TiO_2_@ALP‐NEs (**Figure** **3**a,b). ZGO@TiO_2_@ALP‐NEs showed insignificant antiproliferative effect toward GL261 cells after co‐incubation (less than 20%). While, the cytotoxicity of ZGO@TiO_2_@ALP‐NEs was greatly enhanced upon ultrasound irradiation (more than 95%) (Figure 3c).

**Figure 3:**
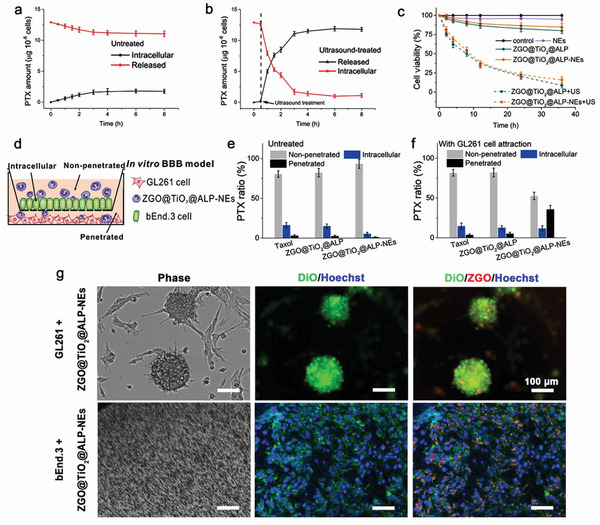
In vitro PTX release from and retained in ZGO@TiO_2_@ALP‐NEs a) without and b) with ultrasound irradiation. The arrows represent the application of ultrasound (5 min, 1.5 W cm^−2^, 1.5 MHz) at the 0.5 h time point. c) Cytotoxicity evaluation against GL261 cells. d) In vitro BBB penetration of ZGO@TiO_2_@ALP‐NEs under tumor attraction. Quantities of PTX were determined in the supernatant (non‐penetrated), endothelial layer (intracellular), and filtrate (penetrated); PTX distribution in the transwell chamber after incubation e) in the absence and f) in the presence of GL261 cells for 6 h. Data are given as mean ± s.d. (*n* = 4). g) Tumor penetration ability of ZGO@TiO_2_@ALP‐NEs. Scale bar, 100 µm. bEnd.3 cell nucleus were stained with Hoechst 33342. The GL261 and bEnd.3 membrane were labelled with DiO (green).

In vitro BBB model was established to evaluate the BBB penetration and GBM targeting ability of ZGO@TiO_2_@ALP‐NEs (Figure 3d). ZGO@TiO_2_@ALP‐NEs showed comparable inflammation‐activated chemotaxis with NEs (Figure S15, Supporting Information) as ZGO@TiO_2_@ALP did not affect the chemotaxis of NEs. In normal condition, Taxol, ZGO@TiO_2_@ALP, and ZGO@TiO_2_@ALP‐NEs had similar PTX distribution in transwell system. While under GBM attraction, increased number of ZGO@TiO_2_@ALP‐NEs penetrated through the BBB for GBM accumulation (PTX content in lower chamber increased from 1.2% to 35.6%), which was much higher than Taxol (from 3.1% to 3.6%) and ZGO@TiO_2_@ALP (from 2.6% to 5.2%) groups (Figure 3e,f). The tumor‐penetration ability of ZGO@TiO_2_@ALP‐NEs was evaluated by a 3D multicellular tumor spheroid model. ZGO@TiO_2_@ALP‐NEs (ZGO, red) distributed into the DiO labelled GBM sphere (green) after 12 h co‐incubation, proving the ZGO@TiO_2_@ALP‐NEs can infiltrate throughout the tumor tissue for comprehensive treatment (Figure 3g).

GBM targeting ability of ZGO@TiO_2_@ALP‐NEs was evaluated in vivo using GL261 tumor bearing nude mouse model. The biodistribution of ZGO@TiO_2_@ALP‐NEs was monitored by optical imaging after intravenous injection (**Figure** **4**a). The autofluorescence background of mouse body was effectively eliminated during detection due to the absence of in situ excitation (Figure S16, Supporting Information). Clear persistent luminescence (PL) was detected after 2 min in vivo excitation with red LED light. Noticeable ZGO@TiO_2_@ALP‐NEs signals distributed all over the mice body at 1 h post material injection. PL signals existed in brain site at 2 h post injection with a time‐dependent intensity increase, indicating the injected ZGO@TiO_2_@ALP‐NEs successfully penetrated through the BBB for GBM accumulation (Figure 4a). In comparison, ZGO@TiO_2_@ALP group did not show signal at brain site under the same condition, proving the GBM cumulation of ZGO@TiO_2_@ALP was realized by NEs delivery. Neutrophils could be attracted by inflammatory factors in the local inflammatory tumor microenvironment for accumulation. The expression levels of CXCL1/KC in the brain and serum of GBM bearing mice were evaluated based on time after tumor implantations (Figure S17, Supporting Information). The pro‐inflammatory cytokine CXCL1/KC showed elevated expression in GBM with time, indicating the brain of the mice gradually changed to inflammatory state after tumor implantation. This inflammatory stress facilitated the migration of the circulating neutrophil delivered nanoplatform (ZGO@TiO_2_@ALP‐NEs) to penetrate through the BBB for inflamed tumor targeting after intravenous injection.

**Figure 4:**
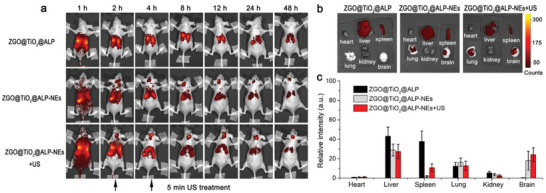
In vivo GBM tracked with ZGO@TiO_2_@ALP‐NEs. a) In vivo persistent luminescence images of GL261 tumor‐bearing nude mice taken at different times post i.v. injection of ZGO@TiO_2_@ALP, ZGO@TiO_2_@ALP‐NEs, and ZGO@TiO_2_@ALP‐NEs with ultrasound treatment (5 min, 1.5 MHz, 1.5 W cm^−2^). b) Ex vivo luminescence images of major organs and brain dissected from mice at 24 h post i.v. injection. c) Semi‐quantitative analysis of ex vivo luminescence images in different organs in (b). Data are presented as means ± s.d. (*n* = 3). The mice were irradiated with the LED light (650 ± 10 nm) for 2 min to activate the persistent luminescence of ZGO core before imaging in (a). The signal acquisition time was 150 s.

The influence of ultrasound treatment on ZGO@TiO_2_@ALP‐NEs accumulation in GBM was explored. Mice were treated with insonation for 5 min at 2 and 4 h post ZGO@TiO_2_@ALP‐NEs injection. ZGO@TiO_2_@ALP‐NEs + US group showed stronger PL signal in brain than ZGO@TiO_2_@ALP‐NEs (Figure 4a). As the ultrasound irradiation at GBM sites initiates ROS generation from ZGO@TiO_2_@ALP, leading to liposome destruction for PTX and anti‐PD‐1 antibody release to kill tumor and induce local inflammation, which in‐turn attractes more ZGO@TiO_2_@ALP‐NEs to migrate into tumor sites for augmented and sustained therapy. Ex vivo imaging and quantification evaluation of the collected organs (24 h post i.v. injection) revealed that only NEs carriage groups realized BBB penetrated material delivery to brain. The ZGO@TiO_2_@ALP‐NEs + US group showed the strongest signal in brain tumors compared with ZGO@TiO_2_@ALP‐NEs and ZGO@TiO_2_@ALP (intensity: 26, 17, and 3, respectively), which was in good agreement with the in vivo imaging (Figure 4b,c).

The administrated ZGO@TiO_2_@ALP mainly accumulated in liver, spleen, lung, and tumor after long time circulations (Figure S18, Supporting Information) and showed limited excretion in vivo (Figure S19, Supporting Information). The ZGO@TiO_2_ carrier could hardly cross the BBB again to return to the blood after liposome destruction. We took histological examinations to verify the potential toxicity of the ZGO@TiO_2_ left in brain (Figure S20, Supporting Information). Negligible material‐induced histopathological changes were found in brains, indicating the long‐term retention of the materials had no harm to brain tissues.

Anti‐GBM efficacy of ZGO@TiO_2_@ALP‐NEs was evaluated on GL261 tumor bearing C57BL/6 mice. The mice were randomly divided into six groups and intravenously injected with saline, Taxol, NEs, ZGO@TiO_2_@ALP, and ZGO@TiO_2_@ALP‐NEs at 21, 24, and 27 days after GBM implantation. In ZGO@TiO_2_@ALP‐NEs + US group, mice were irradiated with ultrasound for 5 min after ZGO@TiO_2_@ALP‐NEs injection (2 and 4 h) to enhance GBM therapy. At 90 days after the initial GBM implantation, the survived mice in ZGO@TiO_2_@ALP‐NEs + US group were implanted with GL261 tumor cells in the other side of the brain for tumor rechallenge (**Figure** **5**a). Histology, survival rate, and brain infiltrating lymphocytes of the treated mice were evaluated for comparison. Histological study showed that, GBM almost disappeared with negligible migration to normal brain tissue after ZGO@TiO_2_@ALP‐NEs + US treatment (Figure 5b). In contrast, aggressive GBM neoplasm still existed with infiltrated borders in saline, Taxol, NEs, ZGO@TiO_2_@ALP, and ZGO@TiO_2_@ALP‐NEs groups (Figure 5b). 40% mice survived in ZGO@TiO_2_@ALP‐NEs + US group at 75 days after tumor implantation, while all mice died in other five groups (Figure 5c). The survived mice behaved normally and showed neglectable change on body weight and pathological changes during therapy (Figures S21 and S22, Supporting Information). GBM re‐implantation only induced 10% death to the ZGO@TiO_2_@ALP‐NEs + US cured mice, while no mouse survived in control group (Figure 5c), indicating ZGO@TiO_2_@ALP‐NEs + US treatment gave long‐term immune‐surveillance toward GBM.

**Figure 5:**
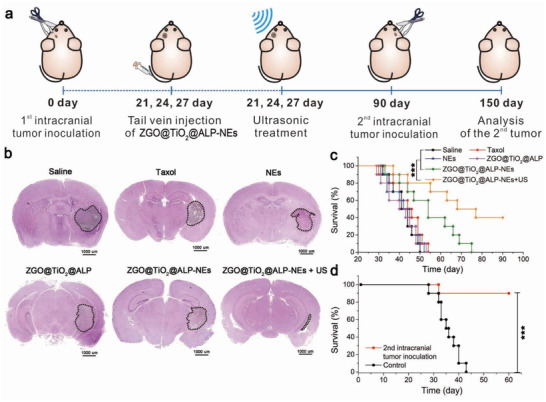
Anti‐GBM effect of ZGO@TiO_2_@ALP‐NEs. a) Experimental schedule for the therapy and long‐term immune‐surveillance against GBM with ultrasound triggered ZGO@TiO_2_@ALP‐NEs. b) Histological study and c) survival analysis of saline, Taxol (3 mg kg^−1^), NEs (5 × 10^6^ cells/mouse), ZGO@TiO_2_@ALP (3 mg kg^−1^ PTX), ZGO@TiO_2_@ALP‐NEs (5 × 10^6^ cells/mouse), and ZGO@TiO_2_@ALP‐NEs + US (5 × 10^6^ cells/mouse, US: 5 min, 1.5 MHz, 1.5 W cm^−2^) treated tumor‐bearing mice. GBM regions were indicated by the dotted lines. d) Survival plot for GBM rechallenged long‐term survivors from (c) ZGO@TiO_2_@ALP‐NEs + US treatment group (*n* = 4) and control mice (*n* = 10). ***P < 0.001, **P < 0.01.

To confirm the long‐term immunological response after the synergistic therapy, mice were sacrificed at days 27 after tumor implantation for GBM infiltrating lymphocytes analysis using flow cytometry (Figure S23, Supporting Information). The CD4^+^FoxP3^+^ regulatory T cells (*T*_reg_) increased significantly in ZGO@TiO_2_@LP‐NEs + US group (57.6%) (only PTX was loaded in the liposome layer in ZGO@TiO_2_@PL‐NEs group) compared with the ZGO@TiO_2_@Liposome‐NEs + US (33.9%), the ZGO@TiO_2_@AL‐NEs + US (30.4%) (only anti‐PD‐1 antibody was loaded in ZGO@TiO_2_@AL‐NEs), and the anti‐PD‐1 antibody treated group (32.7%), indicating the chemotherapy itself resulted in immunosuppression in tumor sites. On the other hand, the addition of the anti‐PD‐1 antibody decreased the percentage of *T*_reg_ from 57.6% (ZGO@TiO_2_@LP‐NEs + US) to 37.3% in ZGO@TiO_2_@ALP‐NEs + US group (Figures S23a,d, Supporting Information). Besides, the percentage of CD8^+^interferon‐*γ* ^+^(IFN‐*γ* ^+^) effect T cells (*T*_eff_) also increased from 54.8% (ZGO@TiO_2_@Liposome‐NEs + US) to 80.2% (ZGO@TiO_2_@AL‐NEs + US) and from 48.3% (ZGO@TiO_2_@LP‐NEs + US) to 71.4% (ZGO@TiO_2_@ALP‐NEs + US) with the addition of anti‐PD‐1 antibody, which was even larger than that of anti‐PD‐1 antibody treated group (52.3%) (Figures S23b, e, Supporting Information). The *T*_eff_/*T*_reg_ value decreased from 2.63 (ZGO@TiO_2_@Liposome‐NEs + US group) to 0.83 in ZGO@TiO_2_@LP‐NEs + US group due to the adverse effects of chemotherapy on immunity. While, after the addition of anti‐PD‐1 antibody, the *T*_eff_/*T*_reg_ value increased from 0.83 (ZGO@TiO_2_@LP‐NEs + US group) to 1.91 in ZGO@TiO_2_@ALP‐NEs + US group (Figure S23f, Supporting Information). The above changes in *T*_eff_ and *T*_reg_ derived from the antitumor immune response facilitation ability of anti‐PD‐1 antibody, largely improved the therapeutic efficiency of PTX and enabled long‐term immune surveillance in GBM therap. Besides, the ZGO@TiO_2_@ALP‐NEs also showed excellent therapeutic ability on the 4T1 tumor‐bearing mouse model in combination with ultrasound assist, which further confirmed the validity of our method (Figure S24, Supporting Information).

In summary, we have realized augmented chemo/immuno therapy against GBM using neutrophil delivered hollow titania covered persistent luminescent nanosensitizer. Neutrophil transportation enabled BBB penetrable material delivery for GBM treatment. The rattle‐type ZGO@TiO_2_@ALP realized sono‐sensitive PTX and anti‐PD‐1 antibody release for specific and augmented GBM therapy. Ultrasound triggered local‐reginal chemo and immunotherapy eradicated the primary GBM and inhibited the metastasis formation, resulting in a significant increase in survival with no overt off target systemic toxicity. Besides, ZGO@TiO_2_@ALP‐NEs with ultrasound irradiation also elicited antitumor immunological memory to prevent tumor recurrence. Taken together, the neutrophil delivered nanosensitizer has the potential to become the effective treatment option for GBM and other cancer treatment.

## Conflict of Interest

The authors declare no conflict of interest.

## Supporting information

Supporting Information
