# Supplementary material for: Neutrophil Delivered Hollow Titania Covered Persistent Luminescent Nanosensitizer for Ultrosound Augmented Chemo/Immuno Glioblastoma Therapy
Source: Adv Sci (Weinh). 2021 Jul 1;8(17):2004381. doi: 10.1002/advs.202004381 (PMC8425909; doi:10.1002/advs.202004381)
Supplement: Supplementary file 1 — Supporting Information [file ADVS-8-2004381-s001.pdf]

WILEY-VCH

© 2021 Wiley-VCH GmbH

ADVANCED SCIENCE Open Access

Supporting Information

for *Adv. Sci.*, DOI: 10.1002/advs.202004381

Neutrophil Delivered Hollow Titania Covered Persistent Luminescent Nanosensitizer for Ultrosound Augmented Chemo/Immuno Glioblastoma Therapy

Yujie Li, Xvcong Teng, Yongji Wang, Chunrong Yang, Xiuping Yan and Jinghong Li**

Supporting Information

Neutrophil Delivered Hollow Titania Covered Persistent Luminescent Nanosensitizer for Ultrosound Augmented Chemo/Immuno Glioblastoma Therapy

Yujie Li, Xvcong Teng, Yongji Wang, Chunrong Yang, Xiuping Yan and Jinghong Li**

Table of Contents

Materials and methods S3

Figure S1. Schematic illustration of ZGO@TiO_2@ALP preparation S14

Figure S2. TEM images of materials S14

Figure S3. XRD patterns of ZGO, ZGO@mSiO_2, ZGO@mSiO_2@TiO_2 and ZGO@TiO_2.. S15

Figure S4. *In vitro* ROS production ability of ZGO@TiO_2 .... S16

Figure S5. Effect of calcination on the ROS generation ability of ZGO@TiO_2 .... S16

Figure S6. N_2 adsorption-desorption isotherms of ZGO@TiO_2. .... S17

Figure S7. Photoluminescence emission spectra of the ZGO, ZGO@mSiO_2, ZGO@mSiO_2@TiO_2 and ZGO@TiO_2 .... S17

Figure S8. Composition stability of ZGO@TiO_2 .... S18

Figure S9. Zeta potential and size distribution of the materials S19

Figure S10. Anti-PD-1 antibody adsorption isotherms to ZGO@TiO_2-NH_2 and ZGO@TiO_2 .... S19

Figure S11. *In vitro* release profiles of PTX from ZGO@TiO_2@ALP at pH 7.4 and 4.5 .... S20

Figure S12. Flow cytometric analysis of the purity of NEs S20

Figure S13. Cytotoxicity of ZGO@TiO_2, ZGO@TiO_2@ALP and PTX towards NEs S21

Figure S14. Cellular uptake of ZGO@TiO_2@ALP by NEs S21

Figure S15. Chemotaxis of ZGO@TiO_2@ALP-NEs and NEs S22

Figure S16. *In vivo* comparison of persistent luminescence imaging S22

Figure S17. Expression of CXCL1/KC in the brain and in the serum of the GBM tumor-bearing mice after tumor implantation S23

Figure S18. Biodistribution of intravenously injected ZGO@TiO_2@ALP-NEs S24

Figure S19. ICP-MS measurement of feces and urine of mice injected with ZGO@TiO_2@ALP-NEs S25

Figure S20. Histological examinations of the brains of the tumor bearing mice S26

Figure S21. Body weight changes of healthy mice after diverse treatment S27

Figure S22. Histological examinations of the main organs of the mice S27

Figure S23. Long-term immune response in brain induced by ZGO@TiO_2@ALP-NEs S28

Figure S24. Therapeutic effect of ZGO@TiO_2@ALP+US in 4T1 tumor model. S29

Materials and methods

Chemicals

All reagents were of the highest available purity and at least of analytical grade. $Zn(NO_3)_2{\cdot}6H_2O$ (99.99%), Ga_2O_3 (99.99%), $Cr(NO_3)_3{\cdot}9H_2O$ (99.99%), tetraethylorthosilicate (TEOS, 98%) and cetyltrimethyl ammonium bromide (CTAB) were purchased from Aladdin (Shanghai, China). Tetrabutyl titanate (TBOT, 97%), anti-PD-1 antibody, cholesterol, stearylamine (SA), paclitaxel (PTX) and N-formylmethionyl-leucyl-phenyl-alanine (fMLP) were purchased from Sigma-Aldrich (USA). 3, 3'-dioctadecyloxacarbocyanine perchlorate (DiO) and Hoechst 33342 was purchased from Beyotime (Shanghai, China). 1,2-dilauroyl-sn-glycero-3-phosphocholine (DLPC) was purchased from Apexbio (USA). 1,2-Distearoyl-sn-glycero-3-phosphocholine (DSPC) was purchased from Echelon (USA). N-formylmethionyl-leucyl-phenyl-alanine (fMLP) was purchased from BioVison (USA). All reagents were used as supplied without further purification.

Methods

Preparation of ZGO@TiO$_2$. Persistent luminescence ZGO nanoparticles were firstly synthesized by hydrothermal method according to previous work.[S1] 0.08 mmol chromium nitrate, 1.79 mmol zinc nitrate and 1.78 mmol gallium nitrate were mixed in 30 mL water to form homogeneous aqueous solution. The pH of the mixture solution was adjusted 7.5 using 30 wt% ammonia solution. After stirred for 2 h at 25 °C, the precursor solution was transferred into teflon-lined autoclave, which was heated at 120 °C for 24 h. The resulting nanoparticles were washed for 3 times with ultra-pure water before lyophilization.

To prepare mesoporous silica coated ZGO (ZGO@mSiO$_2$) 50 mg prepared ZGO nanoparticles and 200 mg CTAB were dispersed in 50 mL NaOH solution (5 mM) under ultrasonication. The mixture was stirred at 45°C. 1.5 mL TEOS solution (10 % v/v, TEOS/methanol) was added dropwise to the mixture. After 90 min reaction, another 1.5 mL TEOS solution (10 % v/v, TEOS/methanol) was added dropwise to the mixture again for reaction. The mixed solution was stirred for 90 min and transferred into teflon-lined autoclave, which was heated at 120 °C for 24 h. After hydrothermal process, the resulting ZGO@mSiO$_2$ was washed for 3 times with ultra-pure water and 2 times with ethanol before lyophilization.

For TiO$_2$ coating, the prepare ZGO@mSiO$_2$ (100 mg), hydroxypropyl cellulose (HPC, 0.1 g) and ultra-pure water (0.15 mL) were dispersed in ethanol (25 mL) under ultrasonication. The mixture was stirred for 30 min at room temperature. 6 mL TBOT (1mL TBOT in 5 mL ethanol) was added to the mixture at a rate of 0.5 mL/min. After 10 min stirring, the temperature of the mixture was increased to 85 °C under refluxing to react for another 100 min. The precipitate was centrifugated and washed with ethanol for 3 times to give ZGO@mSiO$_2$@TiO$_2$.

For SiO$_2$ coating, the above prepared ZGO@mSiO$_2$@TiO$_2$ and polyvinylpyrrolidone (PVP, 0.15 g) were dispersed in ultra-pure water (20 mL) under ultrasonication. After 12 h

stirring, PVP was adsorbed onto the TiO_2 surface. The nanoparticles were separated from solution and dispersed in 5 mL ethanol. 15 mL ethanol (13 mL), 4 mL water, 0.8 mL TEOS and 0.6 mL aqueous ammonia (28%) were sequentially added to the above dispersion. The mixture was stirred for 5 h for SiO_2 coating. The resulting ZGO@mSiO_2@TiO_2@SiO_2 was separated by centrifuge and washed for 3 times with ethanol before lyophilization.

To acquire the rattle-type ZGO@TiO_2, the ZGO@mSiO_2@TiO_2@SiO_2 was calcinated at 800 °C for 2 h to crystalize the amorphous TiO_2 [S2] and enhance the persistent luminescence of ZGO (calcination can generally increase the population of vacancies and facilitate the aggregation of various defects in ZGO crystal[S3]). The calcined ZGO@mSiO_2@TiO_2@SiO_2 were dispersed in 30 mL water and added with NaOH solution (2.0 M, 2 mL) for SiO_2 etching. After stirred for 6 h at 60 °C, the rattle-type ZGO@TiO_2 were isolated by centrifugation and washed for 4 times with water. The acquired ZGO@TiO_2 were finally lyophilized for further applications.

The size distribution and zeta potential of the prepared nanoparticle were measured by Nano-ZS Zetasizer (Malvern, U.K.). Transmission electron microscopy (TEM) images were acquired on the JEM-2010FEF transmission electron microscope (JEOL, Tokyo, Japan). X-ray diffraction (XRD) experiments were performed on a D/max-2500 diffractometer with Cu Kα radiation (Rigaku, Japan). Photoluminescence emission and excitation spectra, and the time decay curves were recorded on F-4500 spectrofluorometer (Hitachi, Japan). BET surface areas data were collected on the NOVA2000e surface area and pore size analyzer (Quantachrome, USA) using N_2 adsorption at 77 K.

Ultrasound triggered reactive oxygen species (ROS) generation of ZGO@TiO_2. The ultrasound triggered ROS generation capability of ZGO@TiO_2 was evaluated using 1,3-diphenylisobenzofuran (DPBF).[S4] Before the experiment, the DPBF acetonitrile solvent was bubbled with air for 30 min. For the ultrasound induced ROS generation evaluation experiments, 2 mL ZGO@TiO_2 (1.0 mg mL^{-1}) was added with 100 μL DPBF (35 μM, dissolved in acetonitrile) and placed in a 10 ml glass vial in dark. The bottle was sealed with latex membrane, smeared with ultrasonic gel and treated with ultrasonic source at determined duration and power (1.5 W cm^2, 1.5 MHz, 5 min). The fluorescence of DPBF at 455 nm was recorded for comparison (ex. 410 nm).

Red LED activated persistent luminescence evaluation. ZGO@TiO_2 were pre-irradiated with red LED light (650 ± 10 nm, 2000 lm) for 2 min. After the cease of LED irradiation, the persistent luminescence of ZGO@TiO_2 was recorded by IVIS Lumina II imaging system (acquisition time 90 s, without excitation). After 60 min recording, the ZGO@TiO_2 was re-irradiated with the same red LED light (650 ± 10 nm 2000 lm) for 2 min and the persistent luminescence was recorded again by the above method.

Preparation of ZGO@TiO_2@ALP. For effective antibody loading, the inorganic ZGO@TiO_2 were firstly functionalized with amino before antibody co-incubation. Briefly, the prepared ZGO@TiO_2 (100 mg) were degassed for 12 h for desiccation, which were subsequently dispersed in anhydrous toluene (2.0 mL) and dropwise added with 28 μL APTES. After refluxed at 60 °C for 12 h, the particles were washed with toluene for 2 times and ethanol for 3 times to remove the unreacted APTES. The amino functionalized ZGO@TiO_2 (ZGO@TiO_2-NH_2) were acquired after 24 h vacuum drying.

To load anti-PD-1 antibody into the nanosensitizer, 15 mg ZGO@TiO_2-NH_2 were put in a 2 mL tube and added with 1600 μL anti-PD-1 stoke (1 mg/mL). The tube was shaken at 1400 min^{-1} under 18 °C for 24 h for antibody incubation. The acquired mixture was centrifugated to remove the unloaded antibody in supernatant. The protein content was

measured by Bradford method (bovine gamma globulin was used as standards). To evaluate the antibody release from ZGO@TiO_2-NH_2, fresh prepared antibody loaded material were dispersed in PBS

To evaluate the *in vitro* anti-PD-1 antibody release from ZGO@TiO_2-NH_2, anti-PD-1 antibody were saturated loaded to 1.0 mg ZGO@TiO_2-NH_2 according to the method described above. 1250 μL PBS was incubated with antibody loaded ZGO@TiO_2@aPD-1 with shaking of 100 rpm. At determined time points, 250 μL of the PBS was drawn out and replaced with an equal volume of fresh PBS. The amounts of released anti-PD-1 antibody were measured at 210 nm using anti-PD-1 antibody as standards.

To encapsulate the prepared ZGO@TiO_2@aPD-1 with paclitaxel (PTX) loaded liposome, 1,2-dilinoleoyl-sn-glycero-3-phosphocholine (DLPC), 1,2-distearoyl-sn-glycero-3-phosphocholine (DSPC), stearylamine (SA), cholesterol and paclitaxel (PTX) (at a molar ratio of 4:3:3:2:1) were firstly dissolved in chloroform methanol mixture (2:1, v:v). The organic solvent was removed by vacuum rotary evaporation (40 °C) to form a thin film. The film was dried under vacuum at room temperature for 12 h and then hydrated with 5 mL 2 mg mL^{-1} former prepared ZGO@TiO_2@aPD-1 PBS solution. The mixed solution was treated with ultrasonic bath (in ice water) and extruded through polycarbonate membrane (with the pore size of 200 nm) for 3 cycles to form the PTX liposome encapsulated nanosensitizer ZGO@TiO_2@ALP.

The PTX loading efficiency and encapsulation efficiency were determined using high-performance liquid chromatography (HPLC) analysis using methanol and purified water as the elute. The PTX encapsulation efficiency was calculated following the formula: (W/W_0 × 100%), W represent for the weight of loaded PTX and W_0 represented for the weight of added PTX. The PTX loading efficiency was calculated by ($W/(W_{all})$ × 100%), where W represent for the weight of loaded PTX and W_{all} represented for the weight of ZGO@TiO_2@ALP. The

final prepared ZGO@TiO_2@ALP had a PTX encapsulation efficiency of 92.6 % with the PTX loading efficiency of 1.2 %.

PTX release was evaluated using dialysis tube. Briefly, 1 mL prepared ZGO@TiO_2@ALP were put into dialysis tube (MWCO = 10 KDa) and immersed into 30 mL PBS buffer (pH 7.4) or HAc-NaAc buffer (pH 4.5) containing 1 % Tween 80 (w:w) for gently shaking (30 rpm, 37°C). At pre-determined time points, 2 mL of the medium was drawn out and replaced with 2 mL fresh medium with the same pH value. PTX content was determined by HPLC. To evaluate the ultrasound triggered PTX release from ZGO@TiO_2@ALP, the prepared ZGO@TiO_2@ALP was irradiated with ultrasound (1.5 W cm^2, 1.5 MHz) for 5 min at the 6 h time point (The dialysed CPIL4 was placed in a 50 mL glass bottle (covered with rubber closure) and the ultrasonic source was conducted to the rubber closure using ultrasonic gel). The released PTX content was measured by HPLC.

Cell lines. Mouse glioblastoma cell line (GL261) and cerebral microvessel endothelial cell line (bEnd.3) were purchased from EallBio Life Sciences Co. Ltd (Beijing, China). GL261 and bEnd.3 cells were cultured in complete Dulbecco's Modified Eagle Medium (DMEM, Hyclone, USA) (supplemented with 10% fetal bovine serum (FBS) (GBICO, USA), 100 U/mL penicillin and 100 μg/mL streptomycin (Hyclone, USA)). The cells were directly used without further authentication after purchase. Neutrophils (NEs) used in the experiments were acquired from the bone marrow of mice. C57BL/6J mice (male, weighing 20 ± 2 g, 6 week) were sacrificed to collect the femur and tibia under aseptic conditions. Cut the ends of the bones and flush the marrow cavity with 1 mL Roswell Park Memorial Institute medium (RPMI-1640, Hyclone, USA) (supplemented with 10% fetal bovine serum) to acquire bone marrow. The acquired marrow was centrifuged for 3 min at 4000 rpm and the precipitated cells were re-suspended with RPMI-1640 for further isolations. The NEs were acquired using the neutrophil isolation kit (Solarbio, Beijing, China) and cultured in RPMI-1640 (containing 10 % FBS) for further applications.

Preparation of NEs loaded ZGO@TiO_2@ALP (ZGO@TiO_2@ALP-NEs). ZGO@TiO_2@ALP were co-incubated with the former isolated NEs to prepare the NEs delivered nanosensitizer (ZGO@TiO_2@ALP-NEs).[S5] The NEs were suspended in FBS free RPMI-1640 medium (2×10^5 cells mL^{-1}) and incubated with ZGO@TiO_2@ALP-NEs at PTX concentration of 100 μg mL^{-1} at 37 °C for 120 min. The prepared ZGO@TiO_2@ALP-NEs were washed 3 times with PBS and re-suspended in PBS for further applications. The ZGO@TiO_2@ALP particles had good dispersions with the out-layer liposome encapsulation and were more likely to sustain in the supernatant under low centrifuge speed. Consequently, after co-incubation, the cell mixture was centrifuged at 1000 rpm for 3 min. The upper 3/4 of the supernatant medium in the tube was gently removed and the sustained ZGO@TiO_2@ALP-NEs in the tube bottom were washed for 4 times according to the same process to wash out the un-internalized nanoparticles. The ZGO@TiO_2@ALP-NEs were fresh prepared for each following experiments. The internalized PTX content in ZGO@TiO_2@ALP-NEs was evaluated by HPLC. The prepared ZGO@TiO_2@ALP-NEs were disrupted to release PTX from the NEs. The lysate was centrifuged at 5000 rpm for 5 min. The supernatant was collected and mixed with methanol for 3 min ultrasound treatment. The mixture was centrifuged at 5000 rpm for 5 min and the PTX content in supernatant was quantified by HPLC.

Cytotoxicity of ZGO@TiO_2@ALP against NEs. The material induced cytotoxicity towards NEs were evaluated using CCK8 kid (Beyotime, Beijing, China). The NEs were seeded in 96 well plates at a concentration of 8000 cells/well and incubated with full 1640 medium for 2 h. Then, the cells were incubated with different concentrations of materials. After 12 h incubation, each well of the plate were added with 10 μL CCK8 solution and further incubated for 30 min. The absorbances of different wells at 450 nm were measured by microplate reader to calculate the cell viability of NEs after treated with different materials.

Physiological functions evaluation of ZGO@TiO_2@ALP-NEs. The inflammation-responsive chemotaxis of ZGO@TiO_2@ALP-NEs was evaluated *in vitro* using transwell migration assay (polycarbonate membrane, pore size: 3 μm, Corning). The transwell chambers were put on the 24-well plate with the FBS-free RPMI-1640 culture medium (containing diverse concentration of N-formylmethionyl-leucyl-phenyl-alanine (fMLP) as chemokines) filled in the lower chamber. ZGO@TiO_2@ALP-NEs, NEs and ZGO@TiO_2@ALP (2×10^5 cells) were added into the upper chamber of the transwell and cultured at 37 °C for 30 min. After the incubation, the number of the cells in lower chambers was counted for comparison. The chemotaxis index was calculated following: $(N_{fMLP} - N)/N$, N_{fMLP} represented for the numbers of cells in the lower chamber after incubation of fMLP added culture medium, N represented for the numbers of cells in the lower chamber without the addition of fMLP.

Ultrasound induced PTX release from ZGO@TiO_2@ALP-NEs. *In vitro* PTX release profiles from ZGO@TiO_2@ALP-NEs were evaluated under normal condition and after ultrasound irradiation (5 min, 1.5 MHz, 1.5 W/cm^2). ZGO@TiO_2@ALP-NEs were cultured in 24-well plates under the concentration of 5×10^5 cells per well with RPMI-1640 medium for pre-determined time period. The PTX content that released from ZGO@TiO_2@ALP-NEs and sustained in ZGO@TiO_2@ALP-NEs were evaluated using HPLC according to the former method.

Cytotoxicity of ultrasound triggered ZGO@TiO_2@ALP-NEs against GL261 tumor cells. *In vitro* antiproliferation efficacy of ZGO@TiO_2@ALP-NEs was tested with ultrasound irradiation and in normal conditions. CCK8 kit was used to measure cell viability after diverse treatments. Briefly, GL261 cells were seeded in 96 well plates (8000 cells per well) for 24 h for cell adherence. NEs, ZGO@TiO_2@ALP, ZGO@TiO_2@ALP-NEs were added to the well plates for different time co-incubation. For the ultrasound treat ZGO@TiO_2@ALP and ZGO@TiO_2@ALP-NEs groups, ZGO@TiO_2@ALP and ZGO@TiO_2@ALP-NEs were pre-

treated with ultrasound for 5 min (1.5 MHz, 1.5 W/cm^2) before be added in to the well plates. The cell viabilities of GL261 cells were measured by CCK-8 kit using microplate reader according to the instruction.

Chemotactic migration of ZGO@TiO_2@ALP-NEs across the in vitro blood-brain-barrier (BBB). *In vitro* BBB model was firstly established to verify the chemotactic migration of ZGO@TiO_2@ALP-NEs. Transwell chambers were coated with gelatine and put in 24-well plates to give a suitable environment for cerebral microvascular endothelial cells growth. bEnd.3 cells were seeded in the upper chamber of the transwell (3 × 10^5 cells/well) and cultured with full RPMI-1640 medium for 48 h to form cell monolayer to simulate the BBB *in vitro*. ZGO@TiO_2@ALP-NEs were added to the upper chamber of the in vitro BBB model. FBS free 1640 medium containing 10 nM fMLP was added in the lower chamber in the wall plates. The systems were incubated at 37 °C for 6 h. The total substances in upper, lower and bEnd.3 cell layer seeded on the transwell were then harvested to measure the PTX content in each part for comparison.

***In vitro* solid tumor penetration ability evaluation.** To evaluate the tumor penetration ability of prepared ZGO@TiO_2@ALP-NEs *in vitro*, 3D tumor spheroid models were established using GL261 cells. 24-well plates were pre-coated with 1640 medium containing 2% sterile agarose to provide a stable environment for tumor cells accumulated growth. GL261 cells were incubated with DiO (5 μM, with green light emission) for 30 min for membrane stain. Then, the cells were seeded into the plate at the concentration of 5000 cells per well using liquid overlay method, which were cultured for 3 days to form tumor spheroids. The acquired tumor spheroids were monitored by inverted fluorescence microscope. Uniform spheroids with compact structure were selected for further applications. To assess the solid tumor penetration ability of ZGO@TiO_2@ALP-NEs, ZGO@TiO_2@ALP-NEs (1 × 10^5 cells) were added to the tumor spheroid containing well and incubated for 8 h. After the incubation, the tumor spheroids were washed for 3 times with PBS to remove the

unpenetrated ZGO@TiO_2@ALP-NEs. The cells were stained with Hoechst 33342 to indicate nuclear site. The images of the ZGO@TiO_2@ALP-NEs penetrated tumor spheroids were obtained by inverted fluorescence microscope.

Animal and glioblastoma model. C57BL/6J mice (male, weighing 20±2 g) were purchased from Weitonglihua Animal Co. Ltd. (Beijing, China) and kept in an SPF condition with standard temperature and humidity in Laboratory Animal Research Center of Tsinghua University. All the animal experiments were approved by the Animal Experimental Ethics Committee of Tsinghua University and performed in accordance with the committee's guidelines on animal handling. The *in situ* glioblastoma model was established by intracranially implantation of GL261 cells (3×10^5 cells/mouse) to the C57BL/6J mice using stereotaxic apparatus (RWD Life Science Co., Shenzhen, China). The brains of the tumor implanted mice were harvested at pre-determined days for haematoxylin and eosin (H&E) staining to exam the growth of the tumors. The mice were ready to use at 21 day after GL261 implantation.

***In vivo* GBM accumulation ability of ZGO@TiO_2@ALP-NEs.** To evaluate GBM accumulation ability of ZGO@TiO_2@ALP, ZGO@TiO_2@ALP-NEs and ZGO@TiO_2@ALP-NEs with ultrasound irradiation, the biodistribution of the intravenously injected ZGO@TiO_2@ALP and ZGO@TiO_2@ALP-NEs was traced by taking advantage of the near-infrared persistent luminescence emission of the ZGO core of the materials. The prepared ZGO@TiO_2@ALP (3 mg kg^{-1} PTX) and ZGO@TiO_2@ALP-NEs (5×10^6 cells/mouse, equivalent to 3 mg kg^{-1} PTX) were irradiated with red LED light (650 ± 10 nm 2000 lm) for 2 min and intravenously injected to the GBM tumor bearing mice. The mice were imaged by IVIS Lumina II imaging system (PerkinElmer) (acquisition time 150 s, without excitation) at different time points to trace the biodistribution of the injected materials. In ZGO@TiO_2@ALP-NEs+US group, the mice were treated with ultrasound irradiation (5 min, 1.5 MHz, 1.5 W/cm^2) on head to trigger PTX release from ZGO@TiO_2@ALP-NEs at 2 h and

4 h post material injection. At 24 h post injection, the main organs (heart, liver, spleen, lung, kidney and brain) of the mice were harvest for *ex vivo* luminescence imaging. Semi-quantitative analyses were conducted by *ex vivo* luminescence imaging to indicate the content of the injected materials in different organs.

***In vivo* GBM therapy assay.** To evaluate the ultrasound augmented therapeutic efficacy of prepared ZGO@TiO_2@ALP-NEs, GBM tumor bearing mice were randomly separated into 6 groups (10 mice per group) named saline, Taxol, NEs, ZGO@TiO_2@ALP, ZGO@TiO_2@ALP-NEs and ZGO@TiO_2@ALP-NEs+US group. The mice were intracranially implanted with GL261 cells to establish *in vivo* GBM model for therapy. At day 21, 24 and 27 post tumor implantation, the tumor bearing mice in different groups were separately administrated with saline, Taxol (3 mg kg^{-1}), NEs (5×10^6 cells/mouse), ZGO@TiO_2@ALP (3 mg kg^{-1} PTX), ZGO@TiO_2@ALP-NEs (5×10^6 cells/mouse) and ZGO@TiO_2@ALP-NEs (5×10^6 cells/mouse) by tail vein injection. In ultrasound treated group, the mice were treated with ultrasound irradiation on head for 5 min (1.5 MHz, 1.5 W/cm^2) at 2 h and 4 h post ZGO@TiO_2@ALP-NEs injection to trigger drug release in GBM sites. The weight and the survival rate of the mice in each group were recorded during the therapeutic process. At 49 days after tumor implantation, one mouse was taken from each group for sacrifice to harvest the brain for histological analyses (H&E staining) to verify the therapeutic outcome in diverse six groups.

Analysis of tumor infiltrating immune cells populations. The mice were sacrificed at day 49 after tumor implantation for brain harvest. The total lymphocytes were extracted from the whole brains and isolated with a 30 %-37%-60% Percoll gradient. Briefly, the fresh harvested brains were cut into small pieces and mixed with PBS solution for homogenization by glass homogenizer without addition of digestive enzyme. The single cell supernatants were collected and added in 30 %-37%-60% Percoll gradient for centrifugation. The lymphocytes, microglia and monocytes were contained in the interface of 37%-60%. The acquired

lymphocytes were added with PMA-ionomycin and Golgi stop for 4 h incubation in cell incubator. The activated Lymphocytes were stained with fluorescence labeled antibodies: anti-CD3-FITC (Abcam), anti-CD8-PerCp/Cy5.5 (Abcam), anti-CD4-APC-H7 (BD Biosciences), anti-IFN-γ-APC (Biolegend) and anti-FoxP3-PE (Abcam) antibodies for flow cytometric analysis to verify the regulatory T cells (Treg) and effector T lymphocytes (Teff).

GBM rechallenge assay. At 90 days after GBM implantation (*i.e.* at 69 days after the initiation of diverse treatments), the survived mice (with normal ethology) in ZGO@TiO_2@ALP-NEs+US group were considered cured, which were re-intracranially injected with GL261 cells (3×10^5 cells/mouse) in the contralateral hemisphere for GBM rechallenge evaluation. The parallel group consisted of the same number of native mice (C57BL/6J mice, male, 5 week), which were injected with GL261 cells (3×10^5 cells/mouse) under the same condition.

Statistical analysis. Experiment results were shown as the mean ± s.d. as indicated. Two-tailed Student's *t*-test was applied to test the statistical significance of difference between two groups. Survival benefit was determined by the Log-Rank (Mantel–Cox) test. The threshold for statistical significance was $*P < 0.05$

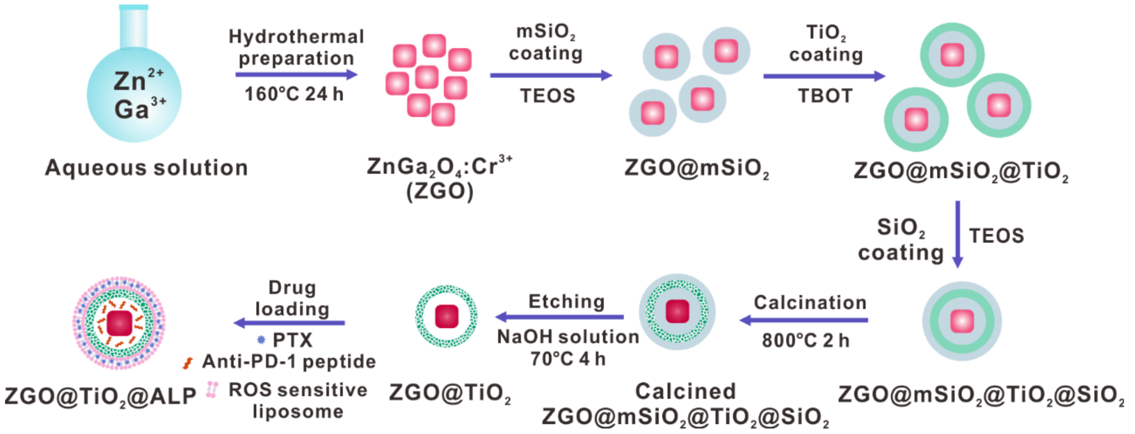

Figure S1. Schematic illustration of ZGO@TiO_2@ALP preparation.

The ZGO@TiO_2 were prepared by silica protection method.[S6] Hydrothermal prepared ZGO core[S1] were coated with mesoporous silica ($mSiO_2$)[S7] to give ZGO@$mSiO_2$, which

were sequentially covered with amorphous TiO_2 and another sacrificial SiO_2 layer with sol-gel method[S6] to prepare ZGO@mSiO_2@TiO_2@SiO_2. To improve the persistent luminescence intensity of ZGO core for *in vivo* optical imaging and transform the amorphous TiO_2 shell to anatase structure for ultrasound triggered ROS generation,[S8] ZGO@mSiO_2@TiO_2@SiO_2 were treated with high temperature calcination. The protective silica layer was finally etched with NaOH solution to acquire the rattle-type ZGO@TiO_2 for further experiments.

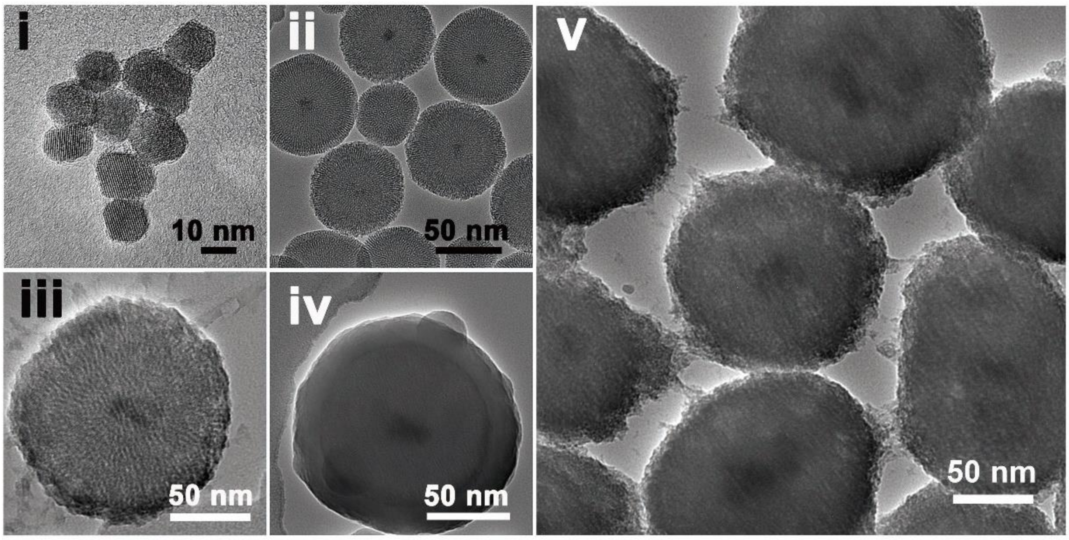

Figure S2. TEM images of **i)** ZGO, **ii)** ZGO@mSiO_2, **iii)** ZGO@mSiO_2@TiO_2, **iv)** ZGO@mSiO_2@TiO_2@SiO_2 and **v)** ZGO@ TiO_2.

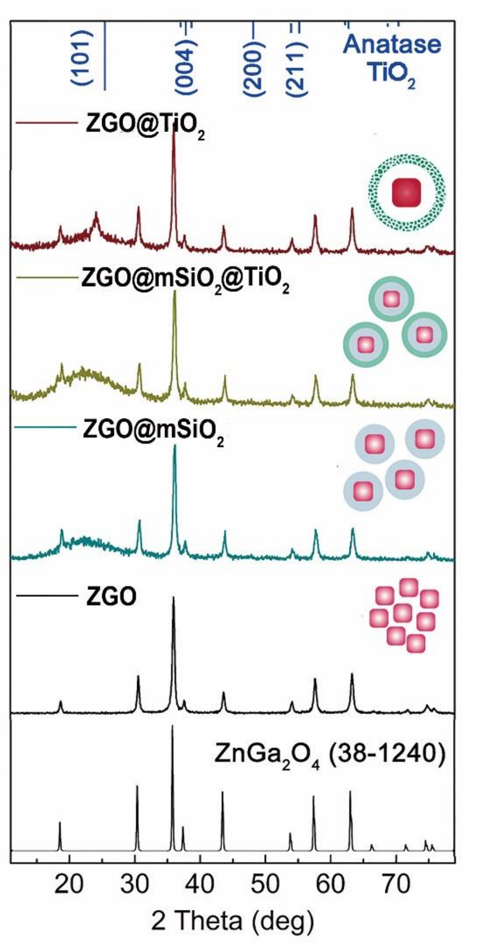

Figure S3. XRD patterns of ZGO, ZGO@mSiO_2, ZGO@mSiO_2@TiO_2 and ZGO@TiO_2.

ZGO@mSiO_2@TiO_2 had a broad peak between 20° and 35° before calcination, indicating amorphous phases for silica and titania. As the protected SiO_2 shells remains amorphous at the calcination temperature, the change in the diffraction peaks relates to the crystalline phase transition of the TiO_2 and ZGO. XRD peaks corresponding to anatase TiO_2 emerged with the high-temperature treatment. After the calcination at 800 °C and the elimination of the SiO_2 layer with NaOH etching, characteristic peaks for anatase TiO_2, especially the peak at $2\theta = 25$ ° (correspond to the (101) crystal planes) was clearly identified. After silica etching process, identical diffraction peaks of mSiO_2 disappeared in ZGO@TiO_2 compared with ZGO@mSiO_2 and ZGO@mSiO_2@TiO_2.

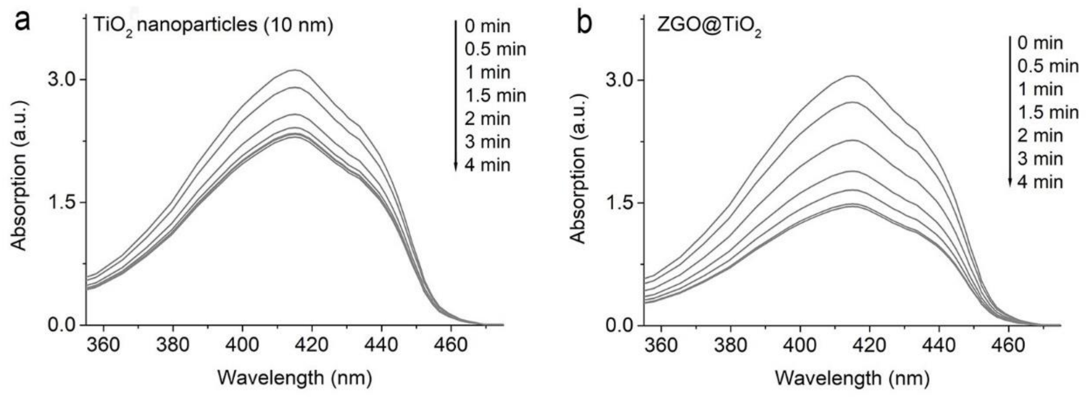

Figure S4. *In vitro* ROS production ability of ZGO@TiO_2. Time-dependent UV-vis absorption change of diphenylbenzofuran upon ultrasound irradiation (1.5 MHz, 1.5 W cm^{-2}) in the presence of **a)** TiO_2 nanoparticles (10 nm) and **b)** ZGO@TiO_2.

In vitro ROS generation of ZGO@TiO_2 and pure TiO_2 nanoparticles (10 nm) under insonation were measured by the fluorescent indicator diphenylisobenzofuran (DPBF) as the UV-vis absorption of DPBF at 410 nm can be quenched by the ROS. The ZGO@TiO_2 possesses higher ROS generation efficiency than the nanosized TiO_2 particles, which was preferred for further applications.

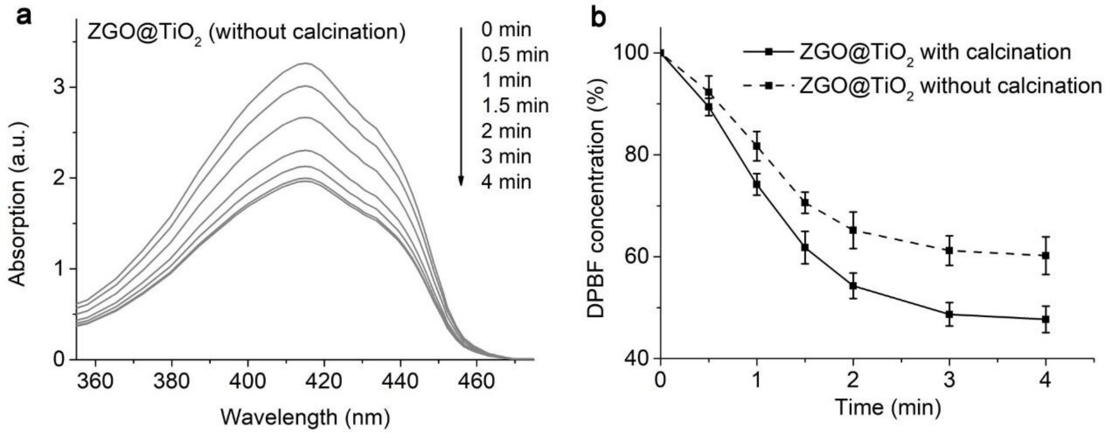

Figure S5. Effect of calcination on the ROS generation ability of ZGO@TiO_2. **a)** Time-dependent UV-vis absorption change of diphenylbenzofuran upon ultrasound irradiation (1.5 MHz, 1.5 W cm^{-2}) in the presence of ZGO@TiO_2 without calcination. **b)** Comparison of ROS generation ability of ZGO@TiO_2 with and without calcination. Data are shown as mean ± s.d. (n = 3).

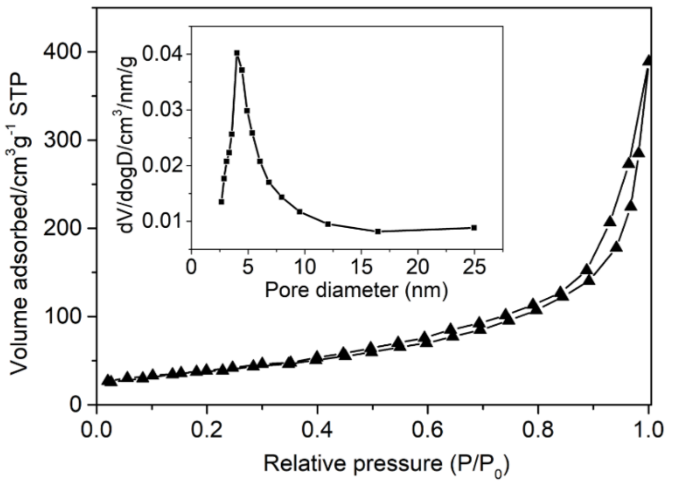

Figure S6. N_2 adsorption-desorption isotherms and corresponding pore diameter distributions of ZGO@TiO_2.

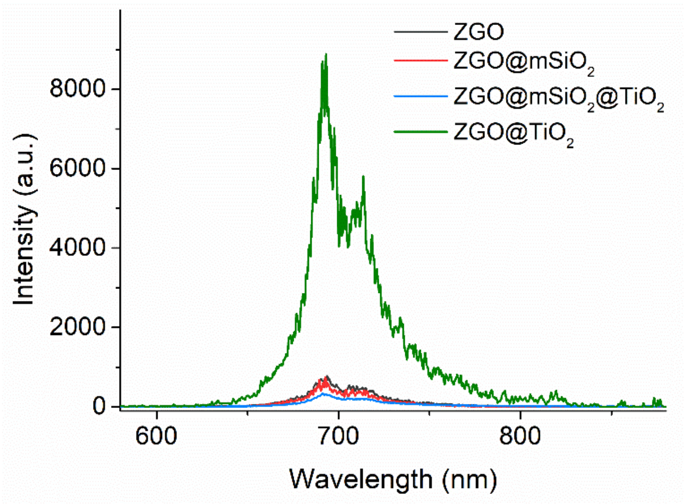

Figure S7. Photoluminescence emission spectra of the ZGO, ZGO@mSiO_2, ZGO@mSiO_2@TiO_2 and ZGO@TiO_2 (λ_{ex} = 254 nm).

ZGO@TiO_2 showed stronger photoluminescence emission than ZGO, ZGO@mSiO_2 and ZGO@mSiO_2@TiO_2 as the calcination treatment remarkably optimized the optical property of ZGO,[S3] which was preferred for high resolution imaging.

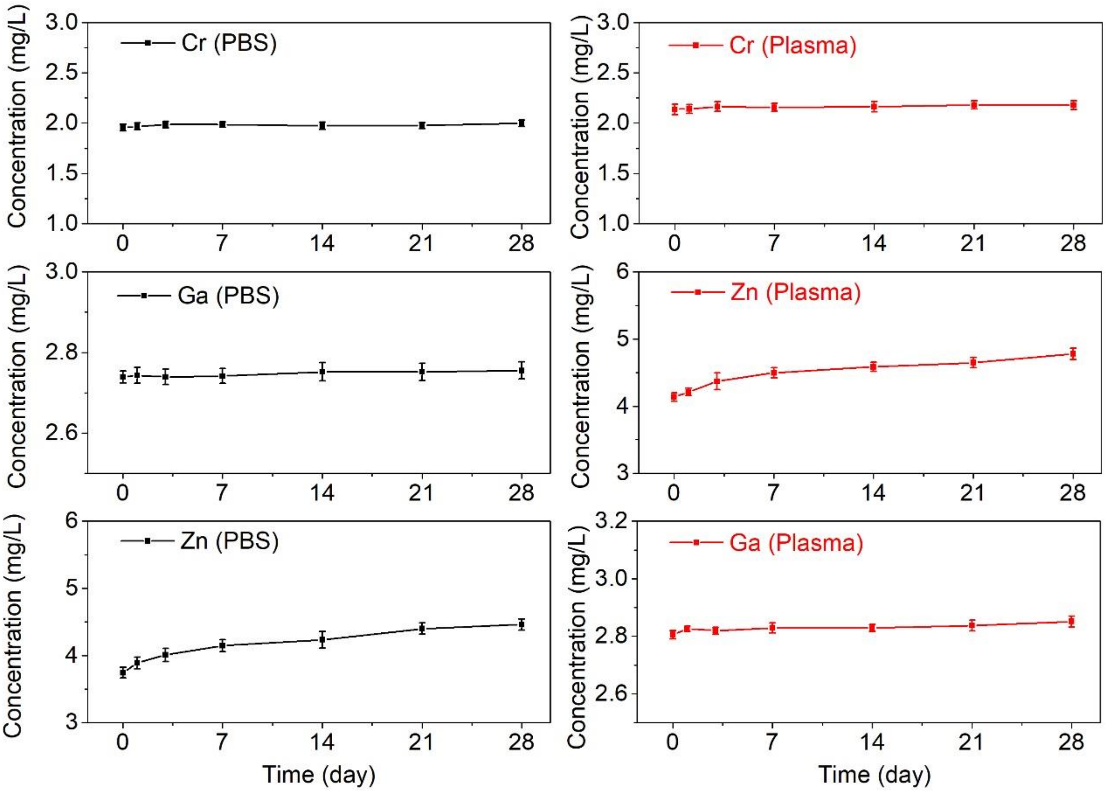

Figure S8. Composition stability of ZGO@TiO_2. Data are shown as mean ± s.d. (n = 4).

ZGO@TiO_2 (1mg/mL) were separately dispersed in PBS (pH 7.4) and plasma (added with heparin to avoid coagulation) for stability evaluation. Supernatant in each group was collected according to the pre-determined time points for ionic concentration evaluation with ICP-MS. Negligible ionic concentration changes were found during 28 days measurements, indicating the biostability of ZGO@TiO_2, which was suitable for *in vivo* applications

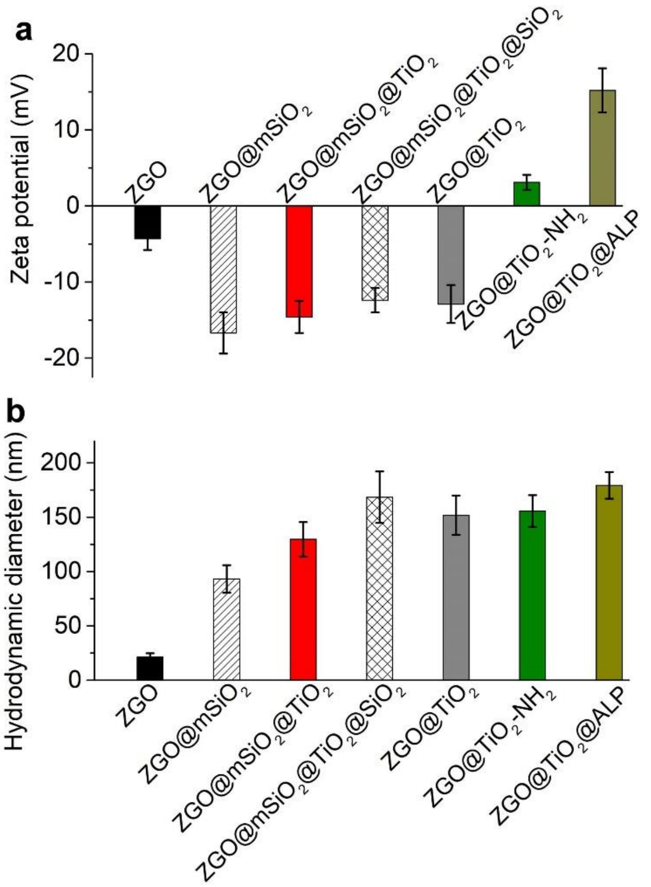

Figure S9. **a)** Zeta potential and **b)** size distribution of the materials. Data are shown as mean ± s.d. (n = 3).

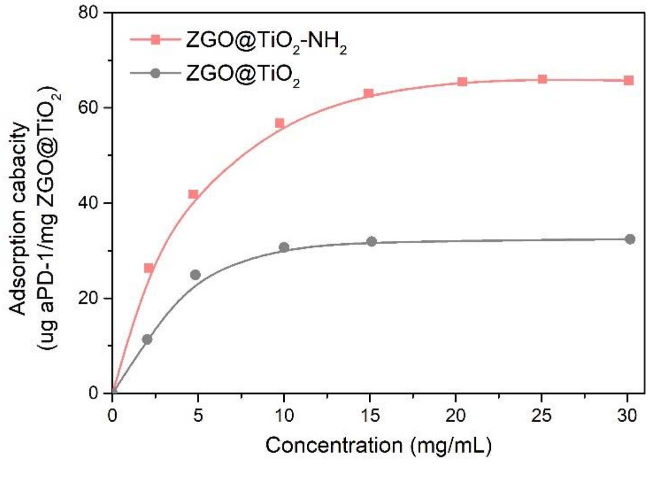

Figure S10. Anti-PD-1 antibody adsorption isotherms to ZGO@TiO_2-NH_2 and ZGO@TiO_2.

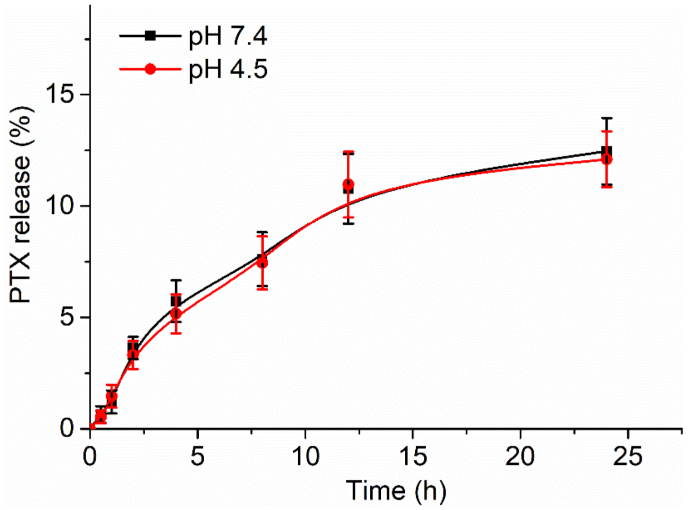

Figure S11. *In vitro* release profiles of PTX from ZGO@TiO_2@ALP at pH 7.4 and 4.5. Data are shown as mean ± s.d. (n = 4).

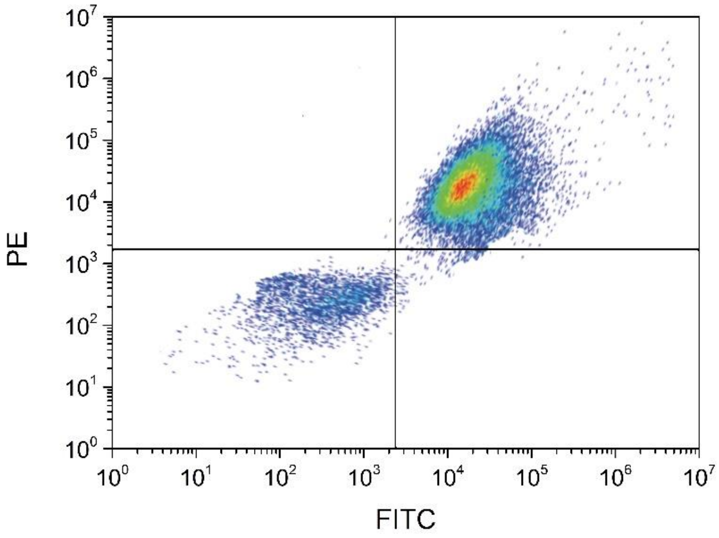

Figure S12. Flow cytometric analysis of the purity of NEs. NEs were stained with Gr-1-FITC and MAIR-IV-PE antibodies.

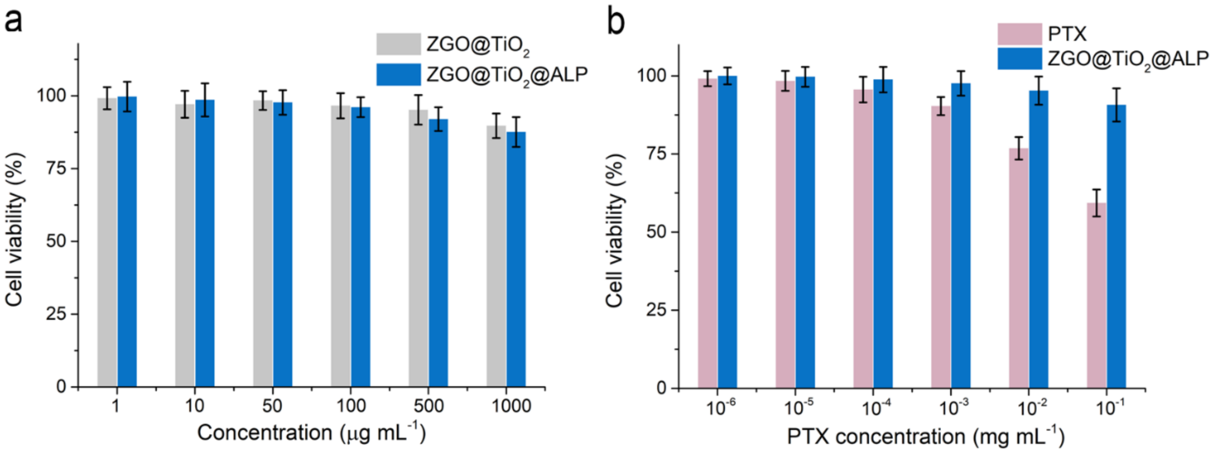

Figure S13. Cytotoxicity of $ZGO@TiO_2$, $ZGO@TiO_2@ALP$ and PTX towards NEs for 12 h. Data are shown as mean ± s.d. (n = 4).

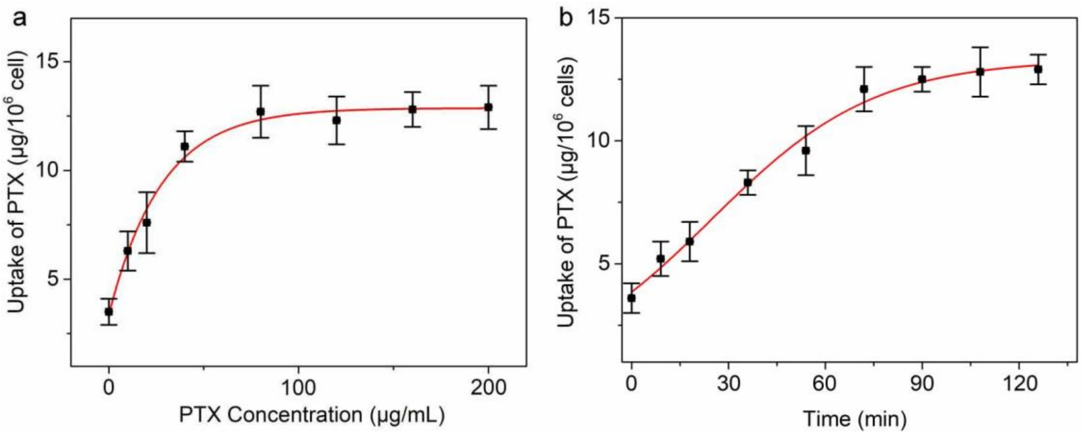

Figure S14. Cellular uptake of $ZGO@TiO_2@ALP$ by NEs. **a**) Quantities of internalized PTX in NEs after 2 h co-incubation of NEs (2×10^5 cells/mL) with different concentration of $ZGO@TiO_2@ALP$. **b**) Quantities of internalized PTX in NEs after co-incubation of NEs (2×10^5 cells/mL) with $ZGO@TiO_2@ALP$ (100 μg/mL representative PTX) for different periods. Data are shown as mean ± s.d. (n = 4 independent experiments).

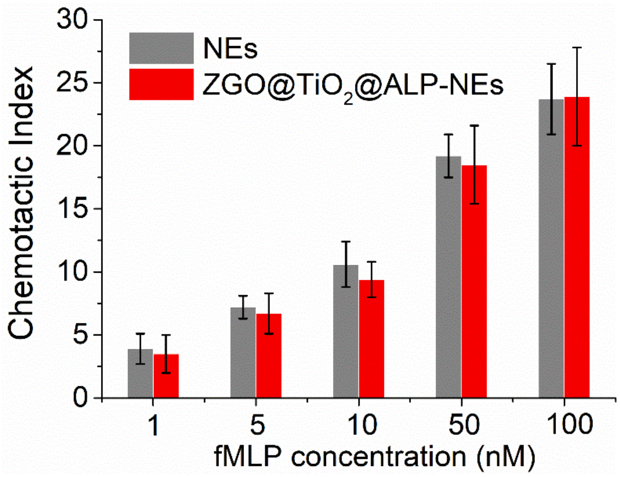

Figure S15. Chemotaxis of ZGO@TiO_2@ALP-NEs and NEs after treatment with different concentrations of fMLP. Data are shown as mean ± s.d. (n = 3).

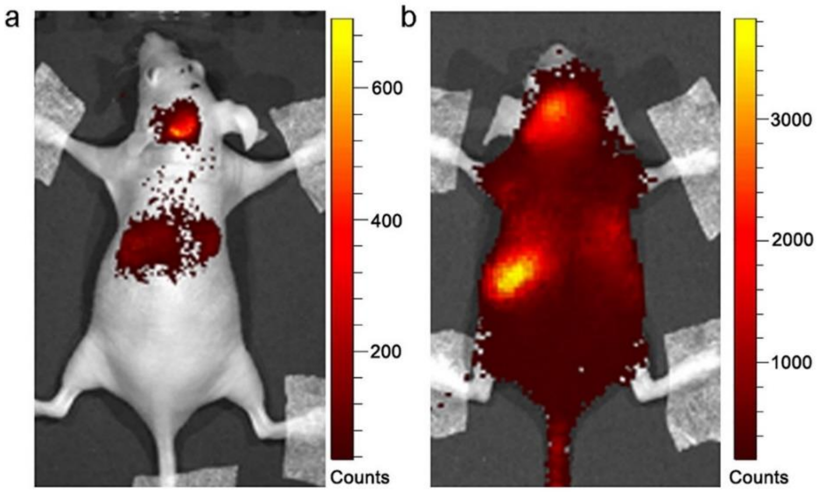

Figure S16. *In vivo* comparison of persistent luminescence imaging of **a**) ZGO@TiO_2@APL-NEs and **b**) fluorescence imaging of Cy5.5 loaded nanoparticle Cy5.5@TiO_2@ALP-NEs at 48 h after intravenous injection GBM bearing mice.

In spite of the clear superiority of Cy5.5@TiO_2@ALP-NEs over ZGO@TiO_2@APL-NEs regarding absolute and total emitted light, *in vivo* detection with persistent luminescence ZGO@TiO_2@APL-NEs not only returns a far better target to background ratio and relative sensitivity, but also ensures real-time imaging of the physiological phenomenon studied *in vivo*.

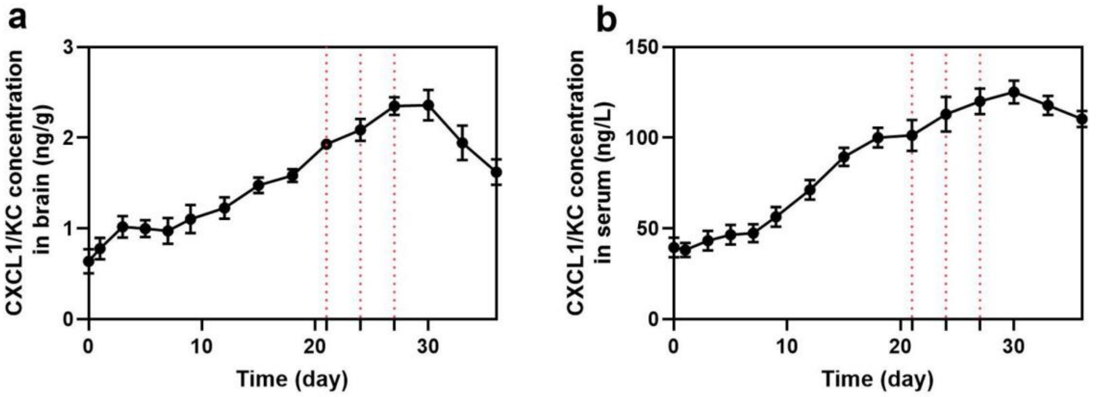

Figure S17. Expression of CXCL1/KC in the **a)** brain and in the **b)** serum of the GBM tumor-bearing mice after tumor implantation. The red dotted lines indicated the point of therapy at 21, 24 and 27 days after tumor implantation. Data are shown as mean ± s.d. (*n* = 3).

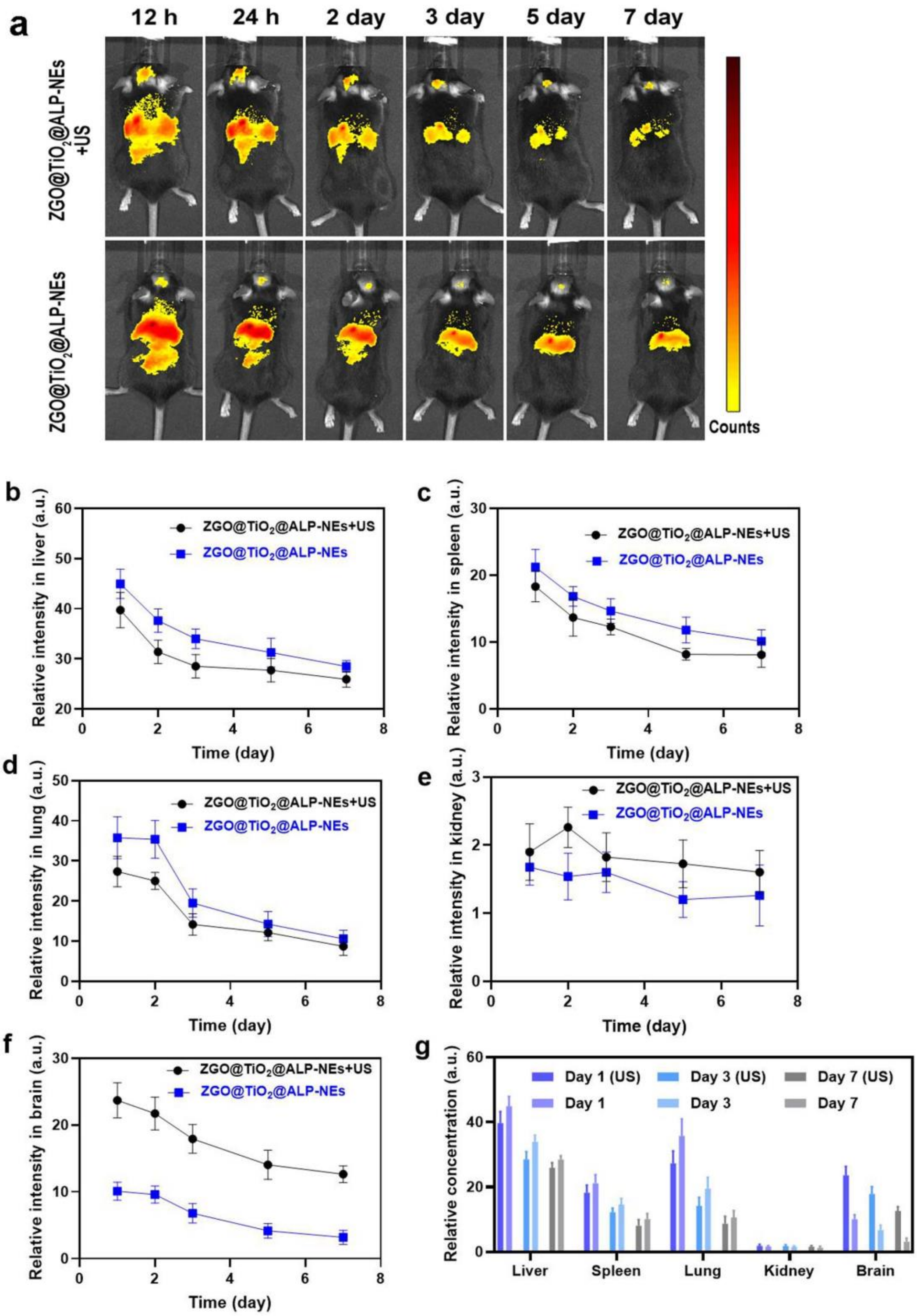

Figure S18. Biodistribution of the intravenously injected ZGO@TiO_2@ALP-NEs. **a)** Persistent luminescence imaging of the tumor bearing mice at different time points after ZGO@TiO_2@ALP-NEs injection. Quantification of ZGO@TiO_2@ALP-NEs in the **b)** liver, **c)** spleen, **d)** lung, **e)** kidney and **f)** brain of the tumor-bearing mice after intravenous administration of ZGO@TiO_2@ALP-NEs (5×10^6 cells/mouse). **g)** Biodistribution of the ZGO@TiO_2@ALP-NEs with and without ultrasound irradiation. The mice were irradiated by

red LED light (650 ± 10 nm, 2000 lm) for 2 min to excite the afterglow of ZGO core before optical imaging. Data are shown as mean ± s.d. (n = 3 independent experiments).

We conducted additional experiments to explore the long-term distribution of the administrated material by optical imaging. The mice were irradiated by red LED light for 2 min to excite the afterglow of ZGO core before optical imaging.

We recorded the optical signals of ZGO@TiO_2 in the main organs to indicate time-dependent distribution of injected materials in tumor bearing mice. Most persistent luminescence signals were found in liver, spleen, lung and tumor after long time intravenous injection of ZGO@TiO_2@ALP-NEs. Negligible signals were found in kidney, indicating the injected material cannot be excreted through the kidney, which was in agreement with the metabolization evaluations. Compared with ultrasound-free group, the ultrasound treatment enhanced the signal in brain and decreased the signals in liver, spleen and lung. ZGO@TiO_2@ALP-NEs showed larger areas under the optical signal curve in ultrasound treated group, demonstrating the ultrasound assistance facilitated the aggregation of ZGO@TiO_2@ALP-NEs in the brain of the GBM-bearing mice.

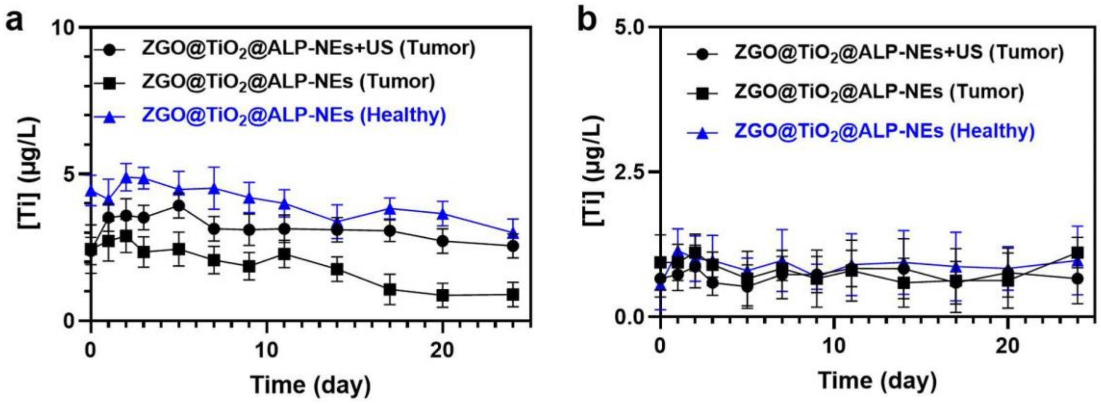

Figure S19. ICP-MS measurement of **a)** feces and **b)** urine of mice injected with ZGO@TiO_2@ALP-NEs (5×10^6 cells/mouse). Data are shown as mean ± s.d. (n = 3 independent experiments).

The time-dependent metabolization of ZGO@TiO_2@ALP-NEs in healthy and tumor bearing mice after intravenous injection was investigated. The feces and urine of the mice

were collected to measure the levels of Ti to determine the whether the ZGO@TiO_2 were cleared via the kidney, intestinal lumen, or bile. Negligible Ti excreted through urine by the kidney both in healthy and tumor bearing mice. Meanwhile, measured levels of Ti excreted from feces also sustained at low levels and showed limited difference in the parallel groups. We therefore speculated that the ZGO@TiO_2 are sequestered and not fully excreted.

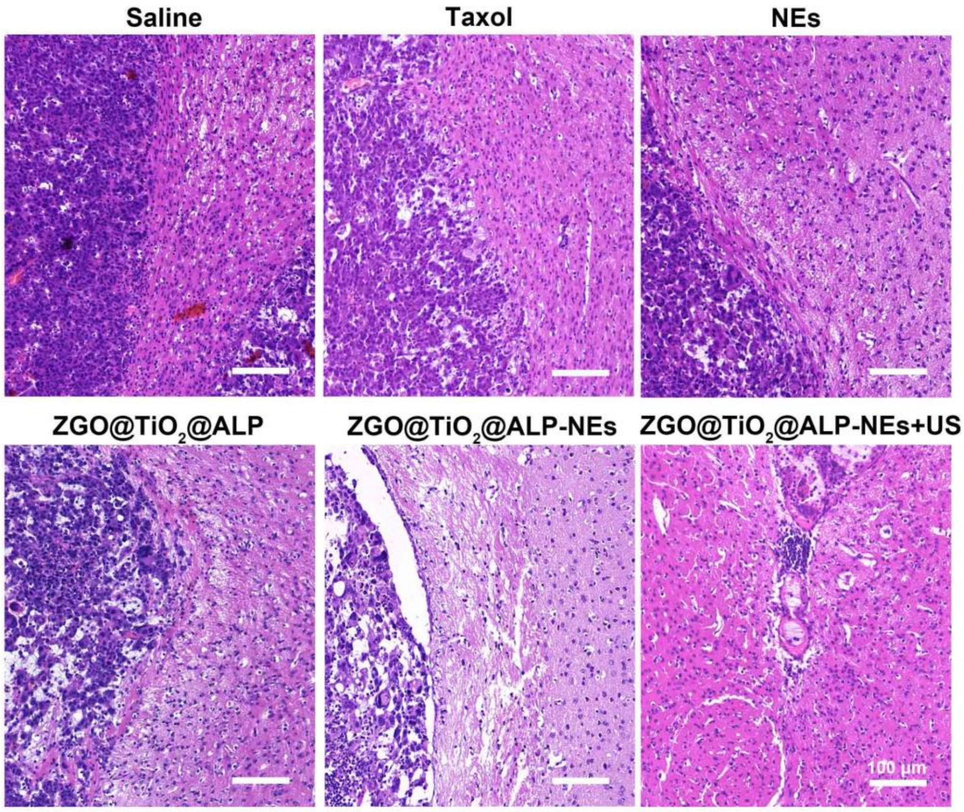

Figure S20. Histological examinations of the brains of the tumor bearing mice after treatment with Taxol (3 mg kg^{-1} PTX), the blank NEs (5 × 10^6 cells/mouse), ZGO@TiO_2@ALP (3 mg kg^{-1} PTX), ZGO@TiO_2@ALP-NEs (5 × 10^6 cells/mouse) and ZGO@TiO_2@ALP-NEs+US (5 × 10^6 cells/mouse). No pathological changes were found after variation after ZGO@TiO_2@ALP-NEs+US treatment. The brains were harvested at 36 days after tumor implantations.

To verify the potential toxicity of the ZGO@TiO_2 left in the brain, we conducted histological examinations of the harvested brains in all groups at 36 days after therapeutic processes and did not find histopathological changes in the normal sites of the brains. This indicated the long-term retention of the materials had no harm to brain tissues.

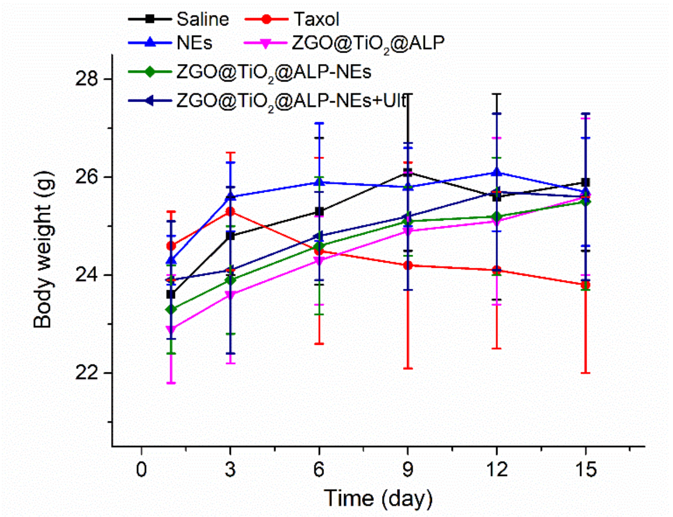

Figure S21. Body weight changes of healthy mice after treatment with saline, Taxol (3 mg kg^{-1} PTX), the blank NEs (5 × 10^6 cells/mouse), ZGO@TiO_2@ALP (3 mg kg^{-1} PTX), ZGO@TiO_2@ALP-NEs (5 × 10^6 cells/mouse) and ZGO@TiO_2@ALP-NEs+US (5 × 10^6 cells/mouse).

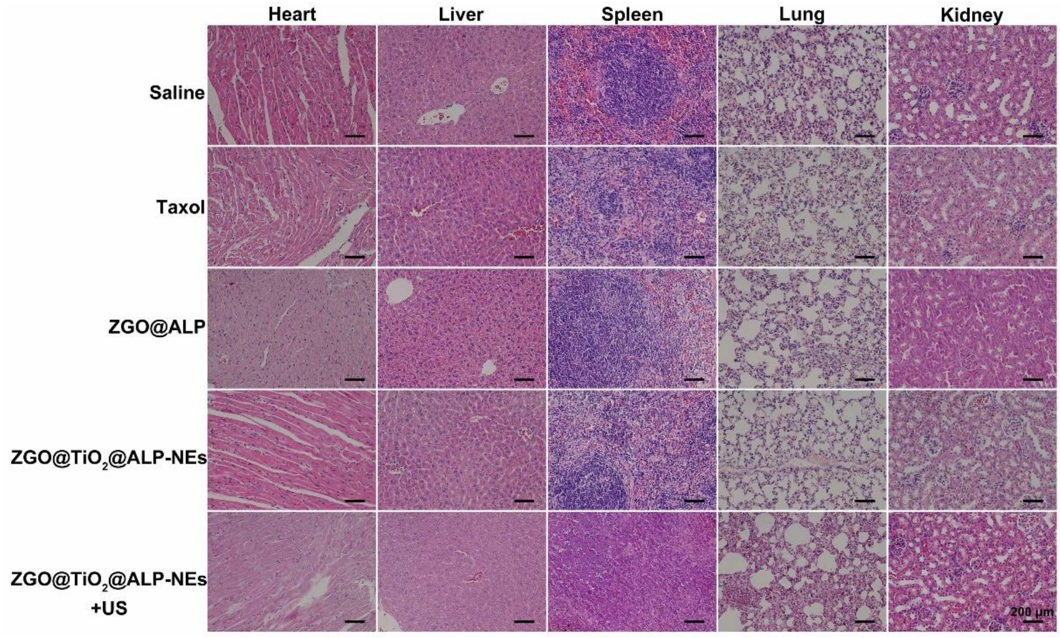

Figure S22. Histological examinations of the main organs of the mice after treatment with Taxol (3 mg kg^{-1} PTX), the blank NEs (5 × 10^6 cells/mouse), ZGO@TiO_2@ALP (3 mg kg^{-1} PTX), ZGO@TiO_2@ALP-NEs (5 × 10^6 cells/mouse) and ZGO@TiO_2@ALP-NEs+US (5 × 10^6 cells/mouse). No pathological changes were found after variation after ZGO@TiO_2@ALP-NEs+US treatment.

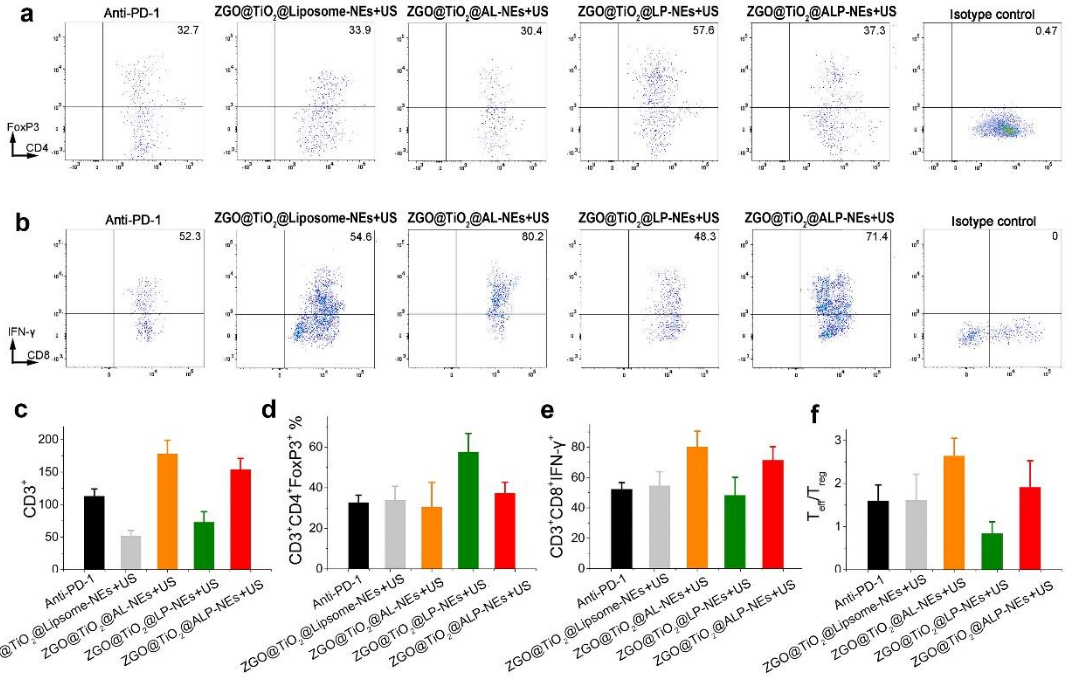

Figure S23. Long-term immune response in brain induced by ZGO@TiO_2@ALP-NEs. **a)** Flow cytometry of the T regulatory cells (T_{reg}) at day 24 post diverse material injection. Flow cytometry plots show results from a single mouse for each group. **b)** Flow cytometry of the T effector cells (T_{eff}) at day 24 post diverse material injection. Flow cytometry plots show results from a single mouse for each group. Quantitive analysis of the composition and proportion of **c)** $CD3^+$ cells, **d)** $CD3^+CD4^+FoxP3^+$, **e)** $CD3^+CD8^+IFN\text{-}\gamma^+$ cells and **f)** T_{eff}/T_{reg} in the brain of the therapeutic groups. Data are presented as means ± s.d. (n = 3).

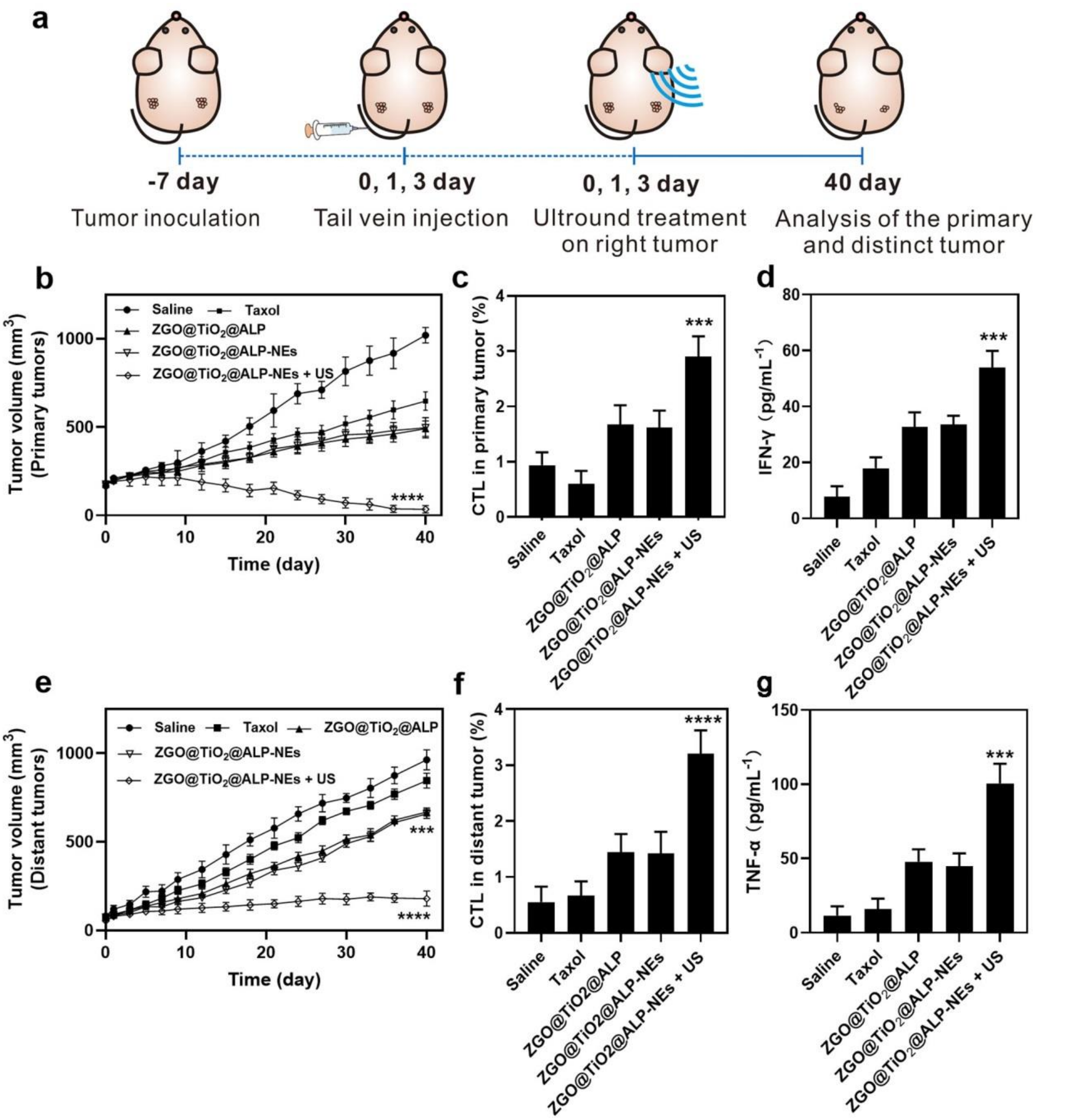

Figure S24. The therapeutic effect of ZGO@TiO2@ALP+US in 4T1 tumor model. **a)** Schematic illustration of ZGO@TiO2@ALP+US therapy. **b)** Primary tumors growth curves of different groups of mice after various treatments. **c)** CTL infiltration in primary tumors of different groups of mice after various treatments. $CD3^+CD8^+$ cells were defined as CTL cells. **d)** IFN-γ concentration after various treatments. **e)** Distant tumor growth curves of different groups of mice after various treatments. **f)** CTL infiltration in distant tumors of different groups of mice after various treatments. **g)** TNF-α concentration after various treatments. ***$p < 0.001$, **$p < 0.01$, or *$p < 0.05$.

We performed additional experiments with a bilateral breast tumor model to validate the therapeutic and long-term immunosurveillance effects of ZGO@TiO_2@ALP-NEs on breast tumors (Please see additional Figure S17, SI).

The bilateral breast tumor model was chosen to simulate the primary and recurrent tumors. To establish the model, 4T1 cells were subcutaneously injected into both the left and right flank regions of mice (Figure S17a). The left and right tumors were designated as the primary and distant (recurrent) tumors, respectively. At 7 days after tumor implantation, the mice were randomly divided into five groups (n = 4 per group) treated with: saline (group 1), taxol (group 2), ZGO@TiO_2@ALP (group 3), ZGO@TiO_2@ALP-NEs (group 4) and ZGO@TiO_2@ALP-NEs with ultrasound irradiation (group 5, ZGO@TiO_2@ALP-NEs + US). Ultrasound irradiation was conducted only on the right tumors (primary tumors) for 5 min at 2 h and 4 h post ZGO@TiO_2@ALP-NEs injection in ZGO@TiO_2@ALP-NEs + US group, whereas the left distant tumors (represented for recurrent tumors) were spared from ultrasound irradiation.

As expected, ZGO@TiO_2@ALP-NEs + US based ultrasound augmented chemo-immuno treatment showed further improved therapeutic efficacy in inhibiting the primary tumor progression in comparison to ZGO@TiO_2@ALP-NEs and other groups (Figure S17b). ZGO@TiO_2@ALP-NEs + US facilitated the infiltration of the cytotoxic T lymphocytes (CTL) in the primary tumor (Figure S17c). The high expression of interferon gamma (IFN-γ) in the serum confirmed the CTL-mediated cellular immunity induced by ZGO@TiO_2@ALP-NEs + US treatment (Figure S17d). Most notably, for tumors on the left side (distant tumors) without ultrasound exposure, the chemo-immuno therapy with ZGO@TiO_2@ALP-NEs + US on primary tumors could also effectively delay their growth with the loaded anti-PD-1 antibody for immunosuppression relief (Figure S17e). Therefore, such ultrasound augmented chemo-immuno treatment with ZGO@TiO_2@ALP-NEs could not only effectively kill

primary tumor cells with direct ultrasound triggered chemo-immuno therapy, but also inhibit the growth of distant tumors for long-term immunosurveillance to avoid tumor recurrence.

To investigate the source for the therapeutic effect on distant tumors in ZGO@TiO_2@ALP-NEs + US group, the cytotoxic T lymphocytes (CTL) infiltration was examined both in primary and distant tumors after therapy. The ZGO@TiO_2@ALP-NEs + US treatment remarkably increased the CTL infiltration in primary and distant tumors compared with other groups (Figure S17c and S17f). We measured the TNF-α in the serum of mice after treatment of primary tumors and found ZGO@TiO_2@ALP-NEs + US induced the highest level of TNF-α in comparison to other groups (Figure S17g). The above results illustrated the ZGO@TiO_2@ALP-NEs + US treatment not only gave therapeutic effects for primary tumor with loaded PTX, but also reverse the immunosuppressive microenvironment inside tumors and triggered T cells for anti-tumor immunities with the delivered anti-PD-1 antibody to kill distant and recurrent tumor cells, which was in consistent with the outcomes in GBM model.

References

[S1] T. Maldiney, A. Bessière, J. Seguin, E. Teston, S. K. Sharma, B. Viana, A. J. J. Bos, P. Dorenbos, M. Bessodes, D. Gourier, D. Scherman, C. Richard, *Nat. Mater.* 2014, *13*, 418.

[S2] J. B. Joo, Q. Zhang, I. Lee, M. Dahl, F. Zaera, Y. Yin, *Adv. Funct. Mater.* 2012, *22*, 166.

[S3] Z. Pan, Y. Y. Lu, F. Liu, *Nat. Mater.* 2012, *11*, 58.

[S4] Q. J. Hu, Y. C. Lu, C. X. Yang, X. P. Yan, *Chem. Commun.* 2016, *52*, 5470.

[S5] J. Xue, Z. Zhao, L. Zhang, L. Xue, S. Shen, Y. Wen, Z. Wei, L. Wang, L. Kong, H. Sun, Q. Ping, R. Mo, C. Zhang, *Nat. Nano.* 2017, *12*, 692.

[S6] I. Lee, J. B. Joo, Y. Yin, F. Zaera, *Angew. Chem. Int. Edit.* 2011, *50*, 10208.

[S7] T. Maldiney, B. Ballet, M. Bessodes, D. Scherman, C. Richard, *Nanoscale* 2014, *6*, 13970.

[S8] S. Yamaguchi, H. Kobayashi, T. Narita, K. Kanehira, S. Sonezaki, N. Kudo, Y. Kubota, S. Terasaka, K. Houkin, *Ultraso Sonoche* 2011, *18*, 1197.
